# Quantitative susceptibility mapping of brain iron in adult ADHD

**DOI:** 10.3389/fpsyt.2026.1735191

**Published:** 2026-02-13

**Authors:** Marcel Schulze, Erik Autenrieth, Behrem Aslan, Rüdiger Stirnberg, Tony Stoecker, Silke Lux, David Coghill, Tim Silk, Alexandra Philipsen

**Affiliations:** 1 Department of Psychiatry and Psychotherapy, University of Bonn, Bonn, Germany; 2 German Center for Neurodegenerative Diseases (DZNE), Bonn, Germany; 3 Department of Physics and Astronomy, University of Bonn, Bonn, Germany; 4 Department of Paediatrics, Faculty of Medicine, Dentistry and Health Sciences, The University of Melbourne, Melbourne, VIC, Australia; 5 Department of Mental Health, The Royal Children’s Hospital, Parkville, VIC, Australia; 6 Neurodevelopment and Disability Research, Murdoch Children’s Research Institute, The Royal Children’s Hospital, Parkville, VIC, Australia; 7 Centre for Social and Early Emotional Development and School of Psychology, Deakin University, Geelong, VIC, Australia; 8 Developmental Imaging, Murdoch Children’s Research Institute, Melbourne, VIC, Australia

**Keywords:** ADHD, brain iron, neurodevelopment, QSM, symptom-coupling

## Abstract

**Background:**

Attention-deficit/hyperactivity disorder (ADHD) is a neurodevelopmental condition that frequently persists into adulthood and is linked to alterations in fronto-striatal circuitry and dopaminergic signaling. Brain iron is essential for dopamine synthesis and neural metabolism and can be indexed *in vivo* using quantitative susceptibility mapping (QSM), which reflects regional magnetic susceptibility. Pediatric studies have mostly reported reduced tissue iron susceptibility in ADHD, but data in adults are limited. This study investigated regional susceptibility in adults with ADHD, its relationship to current and retrospective ADHD symptoms, and depression.

**Methods:**

Twenty-five adults with ADHD and 24 healthy controls underwent 3T MRI, including high-resolution QSM. Mean susceptibility was extracted from 89 cortical and subcortical regions of interest. For each ROI, group effects were estimated using linear regression with heteroskedasticity-consistent (HC3) inference. To account for group differences in depressive symptoms, additional models included Beck Depression Inventory (BDI-II) scores. Dimensional associations with ADHD symptoms were tested using covariate-adjusted ROI-symptom correlations for current symptoms (CAARS) and retrospective childhood symptoms (WURS-k). Benjamini–Hochberg false discovery rate (FDR) correction was applied within each analysis across ROIs.

**Results:**

No ADHD-control group difference survived FDR correction in any model. At the uncorrected level, adults with ADHD showed lower susceptibility in ventral temporal and posterior midline regions, most consistently in the fusiform gyrus and posterior cingulate cortex (small-to-moderate effect sizes across models). When additionally adjusting for depressive symptoms, nominal group differences remained confined to ventral temporal/temporo-limbic and orbitofrontal regions (fusiform, entorhinal cortex, medial orbitofrontal cortex), but again did not survive FDR. Covariate-adjusted ROI-symptom associations did not meet FDR significance; nominally, higher ADHD symptom burden (particularly impulsivity) was associated with lower susceptibility in posterior midline regions (posterior cingulate, precuneus) and ventral temporal cortex (fusiform).

**Conclusion:**

In this adult sample, QSM provided no robust evidence for widespread or regionally specific alterations in brain iron susceptibility in ADHD after multiple-comparison correction. Nevertheless, the reproducible pattern of nominal effects-centered on ventral temporal and default-mode network hub regions-suggests that inter-individual variation in cortical susceptibility may relate to clinical heterogeneity and neurodevelopmental timing rather than constituting a strong diagnostic signature. Larger, medication-stratified and developmentally informed studies are needed to clarify whether subtle iron-related susceptibility patterns track symptom dimensions, treatment exposure, and longitudinal trajectories.

## 1 Introduction

Attention Deficit/Hyperactivity Disorder (ADHD) is a neurodevelopmental disorder with altered levels of attention, and/or hyperactivity/impulsivity (1). Usually considered as a childhood disorder, around 40-60% of children with ADHD show symptoms during adulthood leading to a worldwide prevalence of 2.5% in adulthood (2–4). ADHD has been linked to dysregulation within the brain’s dopamine system, particularly in regions critical for reward processing and executive function, such as the prefrontal cortex and the striatum (5, 6). Altered dopamine transmission in individuals with ADHD is thought to contribute to impairments in motivation, attention, and impulse control (7). This dysfunction is further reflected in structural and functional alterations across key neural networks (8, 9). Functional connectivity studies have consistently shown disruptions in fronto-striatal and fronto-parietal circuits, which are essential for cognitive control (10). Furthermore, individuals with ADHD often exhibit reduced synchronization between the default mode network (DMN) and task-positive network (TPN), which impairs the brain’s capacity to switch between task-focused and resting states (11, 12). The hypoactivity of the dopamine system in ADHD may also lead to compensatory behaviors, such as hyperactivity or impulsivity, as the brain seeks stimulation to normalize dopamine levels (13). The neurotransmitter dopamine is synthesized through a process that depends on iron as a critical cofactor for the enzyme tyrosine hydroxylase (14). This enzyme catalyzes the rate-limiting step in dopamine production, meaning that adequate iron levels are essential for maintaining proper dopamine function. New neuroimaging advancements allow for precise brain iron quantification by applying quantitative susceptibility imaging (QSM) (15). QSM is particularly suited for measuring brain iron because iron is a paramagnetic substance, which alters the local magnetic field and induces changes in magnetic susceptibility detectable via MRI. These susceptibility changes can be quantified and mapped, providing a reliable estimate of iron concentrations in various brain regions. Importantly, QSM offers higher sensitivity and specificity compared to other imaging techniques, such as T2* or R2* relaxation mapping, allowing for more accurate inferences about brain iron distribution (15–17). In children with ADHD, only a handful of studies exist, investigate brain iron by applying various techniques (18). Preliminary evidence points to lower brain iron in children with ADHD, especially in the basal ganglia e.g., Putamen, Globus Pallidum, and Nucleus Caudate (19–23). On the other hand, some studies suggest that differences in brain iron levels between individuals with ADHD and controls are not altered and that variations in brain iron may instead be influenced by comorbid conditions, such as internalizing disorders (24–26). It is worth noting that most studies either did not examine associations with ADHD symptoms or failed to demonstrate significant findings, due to not surviving corrections for multiple comparisons. One study by Shvarzman was able to show a negative relationship between limbic striatal regions and symptom severity on Conners inattentive and hyperactive subscales, whereas Chen et al. reported a linear relationship between left anterior cingulum and symptom severity (19, 20).

As previously mentioned, while ADHD has its origins as a neurodevelopmental disorder, it persists throughout the lifespan, with significant alterations in dopaminergic pathways continuing to manifest in adulthood (3, 27). Recently, the first quantitative susceptibility mapping (QSM) data in adults with ADHD reported regionally increased magnetic susceptibility—interpreted as elevated iron load—in precentral, parietal, cingulate, and striatal territories, and linked higher precentral susceptibility to higher plasma neurofilament light (NfL) (28), suggesting possible neuroaxonal vulnerability and an association with dementia risk later in life. These findings suggest that atypical brain susceptibility is not restricted to pediatric ADHD and raise the possibility that altered susceptibility may have clinical relevance in adulthood. An open question, however, is how regional susceptibility in adults with ADHD relates to core ADHD symptomatology itself. Additionally, it remains unclear (i) whether group differences in susceptibility are detectable in regions supporting attentional selection, salience evaluation, and motivation, (ii) whether such differences are robust to individual variability in depressive symptom severity, which is common in adult ADHD and differed between patients and controls in the present sample, and (iii) whether regional susceptibility scales with current and retrospective ADHD symptom burden. The current study addresses these points by quantifying regional magnetic susceptibility in adults with ADHD and matched controls using QSM. We further examine whether susceptibility in these regions relates dimensionally to current ADHD symptom expression (Conners’ Adult ADHD Rating Scales) and retrospective childhood symptoms (Wender Utah Rating Scale). Based on pediatric evidence of altered striatal susceptibility in ADHD and emerging adult data indicating susceptibility differences in motor and associative cortex, we hypothesize that adults with ADHD would show regionally specific susceptibility alterations relative to controls, and that susceptibility in these regions would covary with ADHD symptom burden.

## 2 Methods

### 2.1 Participants

A total of 25 individuals with ADHD (6 females, mean age: 30.08 years, SD: 9.3) were recruited from the psychiatric outpatient clinic at the university hospital in Bonn, Germany. In addition, 24 healthy controls (9 females, mean age: 26.88 years, SD: 6.3) were recruited through bulletin board advertisements. All participants provided written informed consent, and the study was approved by the local ethics committee (approval number: 166/18). ADHD patients on stimulant medication were asked to discontinue use at least 24 hours before the study. The ADHD diagnosis was established following international guidelines (29, 30) based on the DSM-5 criteria (American Psychiatric Association), with diagnoses made by a specialized psychiatrist following a comprehensive clinical and psychosocial evaluation. This assessment included somatic differential diagnoses, a detailed psychiatric and developmental history, and input from external observers. ADHD symptoms were further evaluated using the Conners Adult ADHD Rating Scales (CAARS) long version, self-rated (31), while retrospective childhood ADHD symptoms were assessed using the Wender Utah Rating Scale (WURS-k) (32). Psychiatric comorbidities were screened using the Structured Clinical Interview for DSM-5 (SCID-5) short interview (33), and depressive symptoms were evaluated through the Beck Depression Inventory (BDI-II) (34).

### 2.2 Neuroimaging protocol

We acquired structural images on a 3.0 T MRI scanner (Magnetom Skyra, Siemens Healthineers) utilizing a 32-channel head coil. Magnetization-prepared rapid gradient echo (MP-RAGE) T1-weighted images were obtained with an acquisition time of 2 minutes and 40 seconds, employing controlled aliasing in parallel imaging results in higher acceleration (CAIPIRINHA) and elliptical sampling (35, 36). The imaging parameters included a repetition time of 2500 ms, an echo time of 3.55 ms, an inversion time of 1100 ms, a flip angle of 7°, a matrix size of 256 × 256 × 176, and a voxel size of 1.0 × 1.0 × 1.0 mm³. The images were acquired in a sagittal slice orientation with a slice parallel imaging acceleration factor of 3, a CAIPI shift of 1, and a Turbofactor of 192.

Magnitude and Phase images were acquired using a rapid, high-resolution 3D echo-planar imaging (EPI) approach that combines CAIPIRINHA parallel imaging with a flexible EPI factor (37). Sequence parameters were: voxel size = 0.7 × 0.7 × 0.7 mm³ isotropic, whole-brain slab-selective binomial-121 water excitation, 210 × 210 × 145.6 mm^3^ oblique-axial field-of-view, echo time 14 ms, repetition time 42.27 ms, 3 × 2_z1_ skipped-CAIPI sampling with an EPI factor of 15 (38), measurement time = 22 s, 8 measurements within 3.5 minutes total acquisition time, including parallel imaging autocalibration. The flip angle was set to 20 degrees. The sequence was performed for both phase-encoding directions (4 measurements with anterior-posterior and posterior-anterior each). Partial Fourier was applied with a factor of 6/8 along the phase encode direction, which was compensated for by weighted averaging in the subsequent preprocessing.

### 2.3 Phase preprocessing and QSM reconstruction

Preprocessing of the phase and magnitude data comprised motion correction (McFlirt) (39). Specifically, motion correction parameters were estimated using a 6-DOF rigid-body model on the magnitude, and the resulting transforms were applied consistently across the corresponding phase/magnitude data to ensure spatial alignment across repetitions and echoes. To correct for residual susceptibility-related warping driven by static B0 inhomogeneity Top-up (FSL) (40) was used, and the resulting warp was applied to the GRE data before reconstruction. Finally, phase matching was performed across repetitions to minimize inter-repetition phase offsets and enable coherent averaging. The corrected phase and magnitude data were then averaged across the eight short acquisitions to improve signal-to-noise ratio and stabilize subsequent susceptibility estimation, as described in (37). QSM maps were reconstructed from the preprocessed images with the Morphology Enabled Dipole Inversion (MEDI) approach (41) as it is implemented in the Ironsmith toolbox (42). In short, MEDI applies phase unwrapping using a region-growing algorithm to correct phase discontinuities and removes background fields through projections onto dipole fields (PDF), ensuring that only the local tissue susceptibility is retained for accurate susceptibility mapping. We visually inspected raw and corrected magnitude/phase images and the resulting QSM maps for motion-related artifacts and verified that rigid-body realignment estimates from MCFLIRT did not indicate problematic motion. Accordingly, no scans were excluded due to motion, which is consistent with the acquisition design, which comprised eight very short measurements (total acquisition time ~3.5 min) using highly accelerated sampling, thereby reducing the likelihood of substantial within-scan motion. After co-registration to T1 images, cerebrospinal fluid (CSF) and white matter (WM) masks were extracted, with refined lateral ventricle CSF masks being applied as a reference structure (susceptibility normalized to zero) to improve the accuracy of susceptibility estimation and to limit artifacts caused by overlapping regions with white matter. QSM-based brain iron concentration values were extracted from 89 gray matter (GM) regions of interest (ROIs) using the AFNI 3dBrickStat function (43). Only positive QSM values, those representing higher magnetic susceptibility than the reference structure, were considered. Any negative values, which may correspond to artifacts from white matter or noise, were excluded from the analysis. Outlier QSM values exceeding the 97th percentile of the positive values within an ROI were also excluded. Following this filtering process, normalized QSM values were computed to account for variability in ROI size across participants.

### 2.4 Statistical analysis

Demographic variables between groups were compared using independent t-tests. To assess brain iron susceptibility differences between ADHD and controls, two linear models were fitted for each ROI (corrected for both age and sex, and age alone): (i) a model with susceptibility as the dependent variable and Group (ADHD vs Control) as the independent variable; and (ii) a model additionally including Beck Depression Inventory (BDI-II) score as a covariate to account for depressive symptom severity, which differed significantly between groups. Parameter estimates for each regional model were obtained using ordinary least squares regression. Inference was based on heteroskedasticity-consistent (HC3) standard errors, from which 95% confidence intervals and Wald-type p-values were derived. For each region of interest (ROI), the group contrast coefficient, its HC3-based 95% confidence interval, and the corresponding p-value were retained for further analysis. False Discovery Rate (Benjamini–Hochberg) correction was then applied across all ROIs per model to obtain q-values. ADHD core symptoms, retrospectively assessed childhood symptoms, age, and depressive symptom severity were analyzed dimensionally across the full sample using partial Pearson correlations with ROI susceptibility. Correlations were adjusted for age and sex (except for the age-ROI association, which was adjusted for sex only) and were corrected for multiple comparisons using FDR.

Given the small sample size, a sensitivity power analysis estimating the minimum detectable effect size (MDES) given the achieved sample sizes and a target power of 0.80 (two-sided α = .05) was conducted. MDES calculations were based on the median complete-case sample size across and are reported for (i) two-sample group comparisons (Cohen’s d), (ii) the unique group term in covariate-adjusted regression models (Cohen’s f²/partial R²), and (iii) correlational analyses (detectable |r|). Under α = .05, the corresponding MDES indicated sensitivity to effects of approximately Cohen’s d ≈ 0.88 for two-sample contrasts, partial R² ≈ 0.16–0.17 for the group term in the primary covariate-adjusted regression models, and |r| ≈ 0.41 for ROI–symptom/ROI–covariate correlations. Full sensitivity results are reported in **Supplementary Table S2**. All analyses were performed in R (version 4.3.1).

## 3 Results

### 3.1 Sample characteristics

For a detailed sample description, see **Table 1**. There was no difference in terms of age and gender. Individuals with ADHD had significantly higher depressive symptoms.

**Table 1:** Sample characteristics.

Demographics	ADHD	Controls
n (female)	25 (6)	24 (9)
Age (SD) in years	30.08 (9.3)	26.88 (6.3)
CAARS, Inattention (SD) **	19.8 (6.6)	6.1 (5.1)
CAARS, Hyperactivity (SD) **	20.29 (8.1)	8.25 (6.1)
CAARS, Impulsivity (SD) **	16.79 (8.8)	4.85 (4)
WURS-k (SD) **	40.42 (11.7)	12.0 (7.74)
BDI (SD) **	9.58 (8.4)	2.3 (2.1)
Past substance abuse	9	–
Lifetime Comorbidities		
Major Depression	5	–
Generalized Anxiety Disorder	4	–
PTSD	1	–
Borderline Personality Disorder	1	–
Medication		
Methylphenidate	5	–
Elontril	1	–
Sertraline	1	–
Triptane	1	–
Gestagen	1	–
L-Thyroxine	1	–

ADHD, attention-deficit/hyperactivity disorder; BDI, Beck Depression Inventory; CAARS, Conners’ Adult ADHD Rating Scale; PTSD, posttraumatic stress disorder; WURS-k, Wender-Utah-Rating-Scale. ** denotes p<.001.

### 3.2 QSM ADHD vs. controls

**Figure 1:**
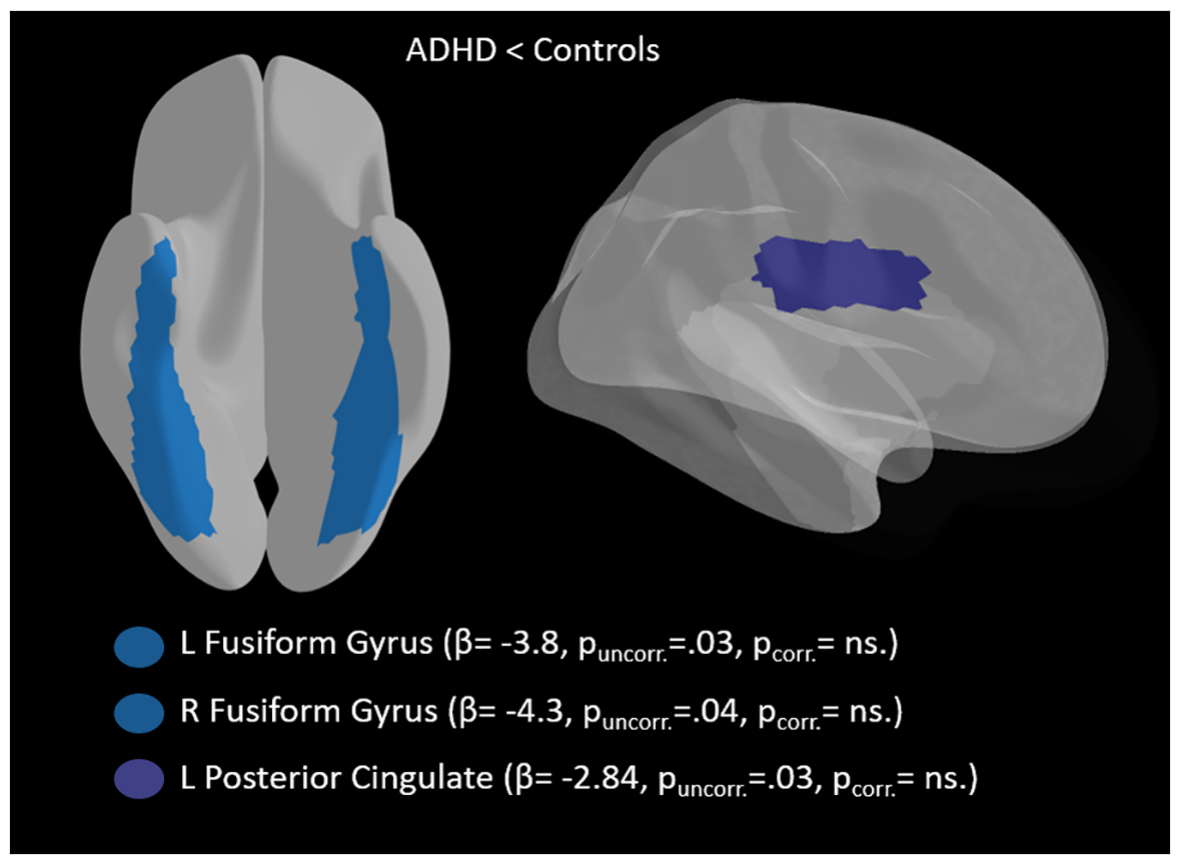
Nominal group differences in brain iron susceptibility between adults with ADHD and controls.

No ADHD–control group effect survived multiple comparison correction. At the uncorrected level, adults with ADHD indicated lower susceptibility relative to controls in the right fusiform gyrus (β = -4.54, 95% CI [-8.65, -0.43], p = .031, q = .96, partial R^2^ =.114) and in the left posterior cingulate cortex (β = -2.84, 95% CI [-5.45, -0.24], p = .033, q = .96, partial R² = .111). In the model where only age was included as a covariate, effects were directionally consistent and of similar magnitude, and additionally implicated the left fusiform gyrus (β = -3.80, 95% CI [-7.30, -0.30], p = .034, q = .93, partial R² = .107), right fusiform gyrus (β = -4.29, 95% CI [-8.36, -0.21], p = .040, q = .93, partial R² = .102), and left posterior cingulate cortex (β = -2.58, 95% CI [-5.13, -0.02], p = .048, q = .93, partial R² = .094). (see **Figure 1**, **Supplementary Table S1**).

In models that additionally adjusted for depressive symptom severity, the overall inference remained unchanged: no effects survived multiple comparisons. The strongest nominal group differences again involved ventral temporal cortex - right fusiform (β = -5.63, 95% CI [-9.94, -1.32], p = .012, q = .67, partial R² = .155) and left fusiform (β = -4.23, 95% CI [-8.14, -0.31], p = .035, q = .67, partial R² = .112), and extended to the left entorhinal cortex (β = -15.15, 95% CI [-27.70, -2.59], p = .019, q = .67, partial R² = .136) and left medial orbitofrontal cortex (β = -7.18, 95% CI [-14.18, -0.17], p = .045, q = .67, partial R² = .102).

### 3.3 Association of brain iron with ADHD symptoms

**Figure 2:**
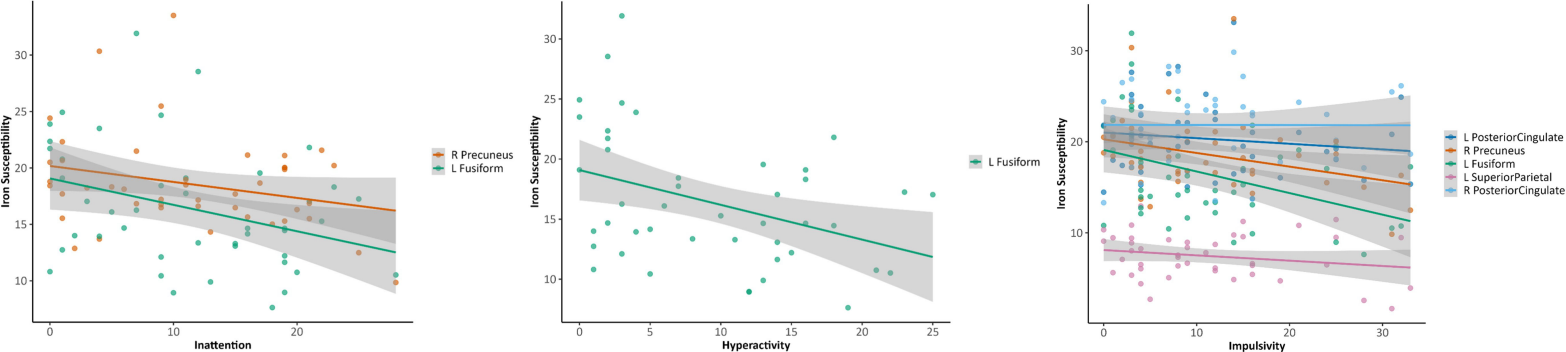
Associations between regional brain iron susceptibility and current ADHD symptom severity (CAARS). Note: These results were nominally significant but did not withstand FDR correction.

**Figure 3:**
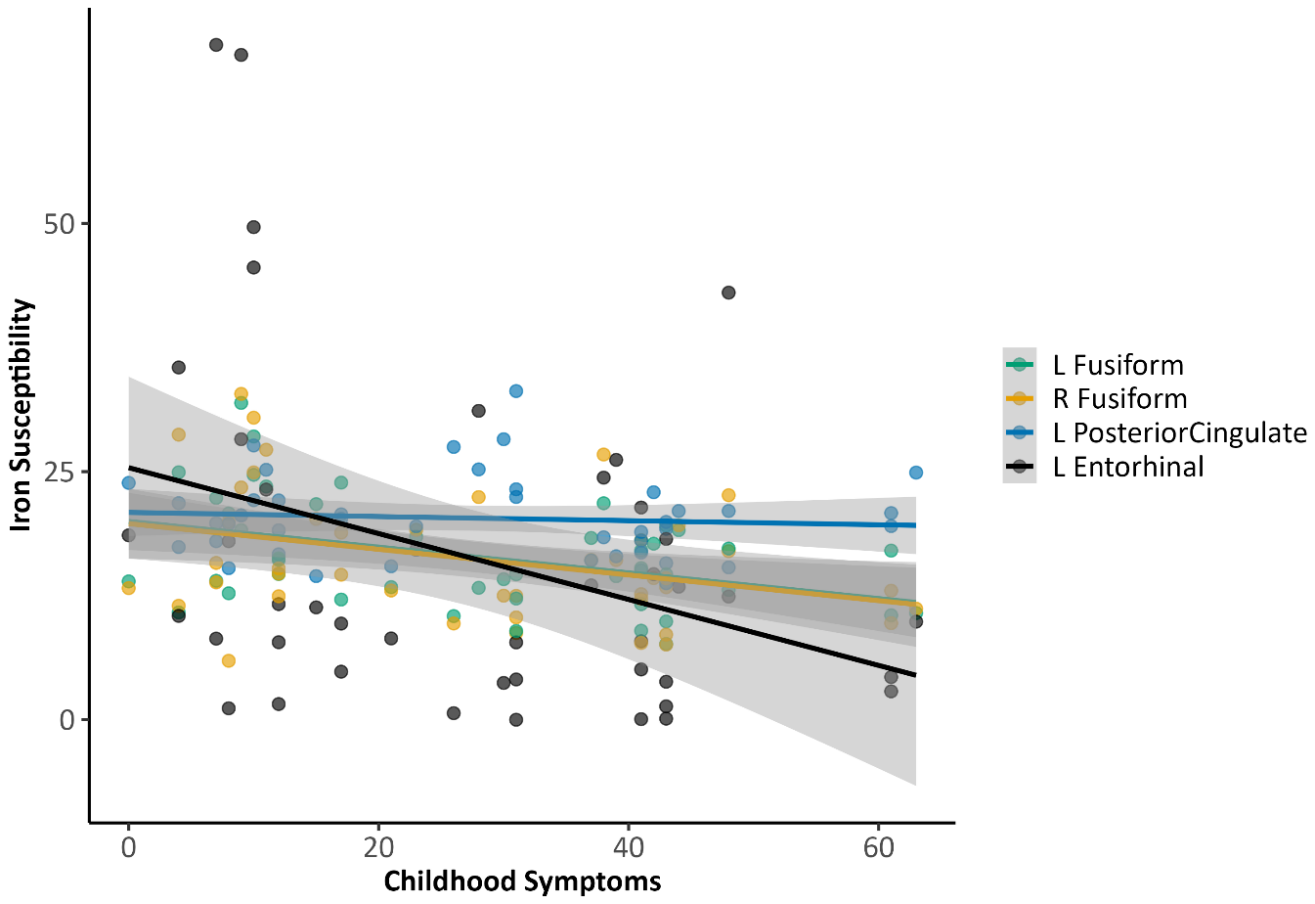
Associations between regional brain iron susceptibility and retrospective childhood ADHD symptoms (WURS-K). Note: These results were nominally significant but did not withstand FDR correction.

Adjusted correlations between iron susceptibility and ADHD symptoms did not meet FDR-significance. At the uncorrected level, higher current ADHD symptom ratings continued to show nominal associations with lower regional magnetic susceptibility in ventral temporal cortex (fusiform gyrus) and posterior midline regions. Specifically, inattention was negatively associated with susceptibility in the right precuneus (r = −0.33, p = .035, q = .88) and left fusiform gyrus (r = −0.31, p = .047, q = .88), while hyperactivity showed a similar negative association with the left fusiform gyrus (r = −0.34, p = .029, q = 1.00). Impulsivity exhibited the strongest nominal pattern, with negative associations in the left posterior cingulate cortex (r = −0.44, p = .004, q = .16) and right precuneus (r = −0.44, p = .004, q = .16), left fusiform gyrus (r = −0.36, p = .021, q = .54), left superior parietal cortex (r = −0.32, p = .041, q = .71), and right posterior cingulate cortex (r = −0.31, p = .048, q = .71) (see **Figure 2**). Retrospective childhood ADHD symptoms (WURS-k) were nominally negatively associated with susceptibility in the left posterior cingulate cortex (r = −0.40, p = .010, q = .69), left fusiform gyrus (r = -0.37, p = .001, q = .69), left entorhinal cortex (r = −0.35, p = .026, q = .69), and right fusiform gyrus (r = −0.32, p = .039, q = .76) (see **Figure 3**).

### 3.4 Association of brain iron and age and depression

Since brain iron levels are not static throughout life, we also examined age-related variations in magnetic susceptibility. After adjusting for sex, no results survived multiple comparison correction, but at the uncorrected level, age was positively associated with susceptibility in posterior cingulate and frontotemporal regions, including the right posterior cingulate cortex (r = 0.41, p = .007, q = .57), left posterior cingulate cortex (r = 0.34, p = .026, q = .80), right superior temporal gyrus (r = 0.33, p = .035, q = .80), and right rostral middle frontal gyrus (r = 0.32, p = .042, q = .80). Depressive symptom severity (BDI) showed nominal negative associations with posterior midline regions (left posterior cingulate: r = −0.36, p = .019, q = .79; right precuneus: r = −0.35, p = .024, q = .79) and a nominal positive association with the bilateral occipital cortex (r = 0.53, p = .036, q = .79).

## 4 Discussion

This study examined regional magnetic susceptibility in adults with ADHD relative to neurotypical individuals using quantitative susceptibility mapping (QSM) and related these measures both to diagnostic group status and to dimensional symptom measures. In contrast to our initial expectations, no ROI survived correction for multiple comparisons, and overall group differences were very subtle. This suggests that global brain iron homeostasis may be largely intact in our adult ADHD sample. Nevertheless, we did observe a few nominal effects that merit discussion. Adults with ADHD showed lower brain iron susceptibility in several cortical regions, notably ventral temporal and limbic areas, relative to controls (e.g., the fusiform gyrus and posterior cingulate cortex). These differences were small and did not survive false discovery rate correction, but their regional pattern is intriguing. The absence of robust case-control differences in brain iron is generally in line with recent findings in younger cohorts. A QSM study of adolescents reported no significant differences in subcortical iron between ADHD and typically developing groups (24), suggesting that alterations of brain iron deficit may not be a consistent feature in adult ADHD. Earlier MRI investigations using iron-sensitive methods did hint at iron abnormalities in ADHD, but those effects appeared tied to developmental stage and treatment exposure. For example, medication-naïve children with ADHD have been found to have significantly lower iron indices in the putamen, caudate, and thalamus compared to controls, whereas children on psychostimulants showed iron levels indistinguishable from healthy (18). This suggests that brain iron differences in ADHD may be most apparent early in the disorder or in unmedicated individuals, and that maturation and/or treatment over time may normalize these differences. By adulthood, many individuals with ADHD could have “caught up” in terms of iron deposition, consistent with the broader pattern of neuroanatomical development in ADHD, where delays in childhood tend to diminish later on (44–46). Our null group findings support the idea that widespread brain iron deficits or excesses are not present in the average adult with well-managed ADHD. In other words, if brain iron dysregulation contributes to ADHD symptoms in youth (perhaps via reduced cofactor availability for dopamine), those effects may be transient or compensated for by adulthood. It is important to note, however, that the literature on adult ADHD brain iron is still sparse and not entirely uniform. Contrary to our results, one recent study reported significantly higher QSM-measured iron in adults with ADHD relative to (28). In that study, the ADHD group showed elevated susceptibility in multiple regions, with the largest effect in the precentral cortex, and a corresponding increase in a blood neuroaxonal injury marker. The authors interpreted this as evidence of excess iron accumulation in ADHD that could render the brain more vulnerable to neurodegenerative processes. In our sample, we did not observe comparable effects: the precentral gyrus and other regions emphasized in that report (e.g., anterior cingulate, precuneus, and basal ganglia) did not exhibit statistically significant case-control differences. In addition to methodological differences, stimulant medication status may have contributed to the discrepant pattern of findings. In the present study, participants were instructed to refrain from ADHD medication (n=5/25) for at least 24 h before MRI acquisition to minimize potential pharmacological effects. In contrast, Berberat et al. reported a naturalistic medication regimen in the ADHD group, with ~59% receiving medication not altered in the context of study participation (28). Although direct evidence in adults is limited, pediatric MRI work suggests that psychostimulant exposure is associated with a relative “normalization” of brain iron indices: medication-naïve youth with ADHD show reduced striatal/thalamic iron markers compared with controls, whereas chronically medicated youth tend to resemble control levels, and longer treatment duration has been linked to a greater degree of normalization (18, 24, 47, 48). Taken together, differences in medication exposure and the clinical characteristics that covary with medication status could plausibly shift regional susceptibility estimates and thereby contribute to the divergence between studies.

In terms of ADHD symptoms, we observed several brain-behavior correlations that, while not significant after controlling for multiple comparisons, suggest potentially meaningful inter-individual variation in susceptibility measures. In particular, susceptibility in the fusiform gyrus showed nominal negative associations with ADHD symptom severity across both current and retrospective indices, such that higher symptom burden tended to relate to lower susceptibility in ventral temporal cortex. Nominal associations were also observed in posterior midline and parietal association regions (precuneus and posterior cingulate cortex, and, for impulsivity, additionally superior parietal cortex), consistent with the involvement of attentional control and default-mode networks in ADHD (49–51). These findings imply that even in the absence of large case-control differences, individual differences in brain iron may track with the severity and history of ADHD. One interpretation is that lower iron in certain cortical regions could be a marker of delayed or altered neurodevelopment associated with more severe ADHD. For instance, the fusiform is involved in higher-order visual processing and attention (52, 53) and prolonged developmental lag or reduced metabolic activity in these regions might manifest as both reduced iron incorporation and greater attentional symptoms. This aligns with the concept that ADHD’s neurobiology may reflect a timing difference in brain maturation rather than a permanent deficit (45, 46). Individuals with the most pronounced or persistent ADHD symptoms might show subtle “fingerprints” of that delayed maturation in measures like QSM.

In exploratory analyses of depressive symptoms, higher BDI scores were associated with lower susceptibility in two default-mode network hub regions-the posterior cingulate cortex and precuneus. In contrast, a positive association emerged in the bilateral occipital cortex. Importantly, none of these correlations survived FDR correction and thus should be interpreted as hypothesis-generating. The direction of the PCC/precuneus associations is nonetheless biologically plausible given (i) the central role of PCC/precuneus within the DMN in depression-related self-referential processing and rumination, and (ii) convergent MRI-QSM evidence in depression showing susceptibility alterations in cingulate regions, including the PCC, in relation to illness course and progression (54–56). From a mechanistic perspective, depression has been linked to dysregulation in iron homeostasis, i.e., iron-related oxidative stress and neuroinflammatory pathways are plausible modulators of regional susceptibility, potentially contributing to subtle DMN-related tissue differences (57, 58).

After adjusting for sex, no age–QSM associations survived correction for multiple comparisons; however, at the uncorrected level, age was positively associated with susceptibility in posterior cingulate and frontotemporal regions. This pattern is consistent with QSM evidence that brain susceptibility tends to increase across adulthood, with particularly robust age effects reported in deep gray matter and more region-specific effects in cortex (59). Although cognitive performance was not the primary focus here, prior work suggests that higher cortical iron/susceptibility can be related to age-associated reductions in fluid cognition and executive functioning, motivating future lifespan studies that jointly model susceptibility and cognition in ADHD (60, 61).

Taken together, our findings paint a nuanced picture of brain iron in adult ADHD. On one hand, the overall null result for case–control differences indicates that altered brain iron levels may not be a defining feature of ADHD in adulthood. The dopaminergic dysfunction in ADHD is more likely attributable to synaptic and receptor-level mechanisms than to a lack of the iron cofactor in dopamine-producing regions. On the other hand, the pattern of nominal effects and correlations we observed hints that brain iron metrics can still yield insight into ADHD’s pathophysiology when examined in detail. The regional specificity of the nominal group differences suggests that subtle microstructural or metabolic differences persist in these circuits. Notably, the posterior cingulate and orbitofrontal cortex are nodes of the default mode and reward networks, which have been reported to be functionally atypical in ADHD (62, 63). If those network differences have a developmental basis, they might leave an imprint on physical tissue properties such as iron content or myelination. Thus, while small, the susceptibility differences in these regions align with the broader understanding of ADHD as involving fronto-limbic and fronto-striatal dysregulation. The fact that these differences did not survive correction emphasizes that they are subtle; yet, they were consistent in direction across multiple analytical models, lending them some credibility. Furthermore, the clinically relevant coupling between susceptibility and symptom burden that we found supports the biological plausibility of our measures. It suggests that QSM, beyond group comparisons, may capture meaningful variation related to ADHD’s clinical heterogeneity.

Brain iron estimated has been proposed as a candidate biomarker for ADHD because iron is tightly coupled to dopaminergic function and because MRI-based approaches provide an *in vivo*, regionally specific readout of tissue susceptibility that may be more sensitive to neurobiological variation than peripheral iron indices (18, 23). In pediatric ADHD, converging evidence suggests that altered brain iron may be detectable and potentially clinically informative, although larger longitudinal studies are required, and the specificity relative to other neurodevelopmental disorders remains unresolved. At the same time, several considerations temper the plausibility of QSM-derived brain iron as a diagnostic biomarker in adults with ADHD. First, adult brain iron is strongly age-dependent and influenced by cumulative exposures (including long-term stimulant treatment histories, lifestyle factors, and cardiometabolic or inflammatory processes), complicating the definition of stable, diagnostically useful thresholds across heterogeneous adult cohorts. Second, in the present data, ADHD-control differences were limited to nominal effects that did not survive multiplicity correction and were sensitive to covariate specification (e.g., adjustment for depressive symptoms), arguing against a robust case-control signature suitable for diagnostic classification. Third, the regional pattern observed here diverges from the canonical pediatric basal-ganglia-centric biomarker narrative, raising the possibility that adult susceptibility differences reflect downstream or compensatory processes, developmental “catch-up,” or comorbidity-related mechanisms rather than a core, disorder-specific marker. Another important interpretive consideration is that associations between QSM-derived susceptibility and lifetime ADHD burden may plausibly reflect bidirectional processes. Brain iron estimates show pronounced age dependence across adulthood and may index multiple microstructural contributors beyond iron, complicating a static trait interpretation in adult cohorts (64). From a consequence perspective, ADHD-related behavioral and clinical exposures (e.g., chronic sleep disruption, stress, and metabolic/inflammatory comorbidity) could influence iron homeostasis and redox-related biology over time. Converging literatures link sleep-wake disturbances and inflammatory-metabolic states to iron regulation and iron-related pathways (65). Conversely, inter-individual differences in brain iron-related susceptibility may contribute to symptom persistence by modulating dopamine-relevant neurobiology and network efficiency, consistent with the broader rationale for investigating brain iron as a candidate biomarker in ADHD, particularly in pediatric work, while acknowledging that adult heterogeneity and cumulative exposures may weaken diagnostic specificity.

### 4.1 Limitations

Several limitations should be considered. First, the study was cross-sectional and primarily correlational, precluding causal inference regarding whether altered regional susceptibility reflects antecedent neurobiological mechanisms contributing to ADHD, downstream consequences of chronic symptomatology and associated lifestyle factors, or compensatory adaptations across development. Bidirectional influences are plausible (e.g., ADHD-related behavioral patterns affecting iron metabolism and, conversely, iron-related neurobiological variation influencing ADHD-relevant circuitry), and disentangling these pathways will require longitudinal sampling and/or quasi-experimental designs that can isolate temporal precedence. Second, the sample size was modest relative to the breadth of regional testing, and findings were exploratory: effects were predominantly nominal and did not survive correction for multiple comparisons, increasing the risk of both false positives and false negatives. Power analyses indicated that the study had ~80% power to detect medium-to-large between-group effects. Under stringent multiple-comparison control across ROIs, only large effects would be detectable, such that smaller effects may have remained undetected despite being potentially meaningful. Accordingly, the present pattern should be interpreted as hypothesis-generating and in need of replication in larger, independently collected datasets. Third, ADHD is clinically heterogeneous in terms of comorbidities that may contribute to variability in susceptibility estimates. These relationships were not fully explored in the context of comorbid psychiatric conditions. Fourth, QSM provides an indirect estimate of iron-related tissue susceptibility and can be influenced by other microstructural sources of magnetic susceptibility, limiting direct biological specificity. Relatedly, ROI-averaged cortical measures may obscure subregional or layer-specific effects and may be sensitive to partial-volume artifacts. Finally, we did not include concurrent systemic iron markers or detailed nutritional/inflammatory measures, which would be valuable to evaluate peripheral-central coupling and to test mechanistic models linking brain susceptibility to iron homeostasis. Although serum ferritin and brain iron levels may not always show a direct correlation in individuals with ADHD, these factors could still have influenced our results (26). Collectively, these limitations motivate larger longitudinal studies integrating detailed clinical trajectories and peripheral iron indices to evaluate the robustness and translational relevance of brain iron measures in adult ADHD, including their potential value for prognosis and treatment-response prediction.

## 5 Conclusion

Overall, this study provides no robust evidence for altered brain iron susceptibility in adults with ADHD. No case-control differences survived multiple comparison correction. At the nominal (uncorrected) level, ADHD was associated with lower susceptibility in a small set of regions, most consistently the right fusiform and left posterior cingulate cortex. While these patterns are neurobiologically interpretable (e.g., higher-order ventral visual/temporo-limbic and DMN-related hubs), they should be treated as exploratory. Collectively, the findings support a cautious interpretation: if QSM differences in adult ADHD exist, they are likely small-to-moderate and regionally nuanced, and future work will require larger, medication-stratified, developmentally informed samples to clarify whether subtle iron-related signatures track symptom dimensions, treatment exposure, and neurodevelopmental timing.

## Data Availability

The original contributions presented in the study are included in the article/**Supplementary Material**. Further inquiries can be directed to the corresponding author.
