# Supplementary material for: Quantitative susceptibility mapping of brain iron in adult ADHD
Source: Front Psychiatry. 2026 Feb 13;17:1735191. doi: 10.3389/fpsyt.2026.1735191 (PMC12946022; doi:10.3389/fpsyt.2026.1735191)
Supplement: Supplementary file 1 [file Table1.docx]

Supplementary Table S1. ROI-wise QSM group-comparison models (adult ADHD vs controls)

Notes. Each row reports the ADHD minus Control group coefficient (β) from ROI-wise linear models of QSM iron susceptibility. Negative β values indicate lower susceptibility in the ADHD group relative to controls. Models: TE_adj = Group + Age + Sex; TE_age = Group + Age; DE_BDI_adj = Group + BDI + Age + Sex; DE_BDI_age = Group + BDI + Age. 95% CIs are based on heteroscedasticity-consistent (HC3) robust standard errors. p denotes the uncorrected two-sided p-value for the group term. q denotes Benjamini–Hochberg false-discovery-rate (FDR) correction. PartialR2 denotes the incremental (semi-partial) R² attributable to the group term in the corresponding regression model. g denotes Hedges’ g (small-sample corrected standardized mean difference) derived from the model-adjusted group effect.

| **ROI** | **Model** | **Direction** | **beta** | **CI** | **p** | **q** | **partialR2** | **g** |
| --- | --- | --- | --- | --- | --- | --- | --- | --- |
| LR_Accumbens_area | TE_adj (Group+Age+Sex) | ADHD < Control | -5.24 | [-20.73, 10.25] | .498 | .967 | 0.012 | -0.22 |
| LR_Accumbens_area | TE_age (Group+Age) | ADHD < Control | -4.86 | [-20.25, 10.54] | .527 | .938 | 0.010 | -0.21 |
| LR_Accumbens_area | DE_BDI_adj (Group+BDI+Age+Sex) | ADHD < Control | -8.86 | [-27.50, 9.78] | .342 | .784 | 0.024 | -0.37 |
| LR_Accumbens_area | DE_BDI_age (Group+BDI+Age) | ADHD < Control | -8.58 | [-27.23, 10.06] | .357 | .809 | 0.022 | -0.37 |
| LR_Amygdala | TE_adj (Group+Age+Sex) | ADHD < Control | -0.16 | [-9.49, 9.18] | .973 | .996 | 0.000 | -0.01 |
| LR_Amygdala | TE_age (Group+Age) | ADHD > Control | 0.40 | [-8.29, 9.09] | .926 | .962 | 0.000 | 0.03 |
| LR_Amygdala | DE_BDI_adj (Group+BDI+Age+Sex) | ADHD < Control | -4.35 | [-14.60, 5.91] | .396 | .784 | 0.019 | -0.34 |
| LR_Amygdala | DE_BDI_age (Group+BDI+Age) | ADHD < Control | -3.93 | [-13.93, 6.07] | .431 | .809 | 0.016 | -0.31 |
| LR_Caudate | TE_adj (Group+Age+Sex) | ADHD > Control | 1.35 | [-7.56, 10.26] | .761 | .970 | 0.002 | 0.10 |
| LR_Caudate | TE_age (Group+Age) | ADHD > Control | 2.19 | [-6.40, 10.79] | .609 | .938 | 0.007 | 0.16 |
| LR_Caudate | DE_BDI_adj (Group+BDI+Age+Sex) | ADHD > Control | 1.52 | [-7.32, 10.35] | .730 | .923 | 0.003 | 0.11 |
| LR_Caudate | DE_BDI_age (Group+BDI+Age) | ADHD > Control | 2.21 | [-6.67, 11.10] | .617 | .840 | 0.006 | 0.16 |
| LR_Hipp | TE_adj (Group+Age+Sex) | ADHD > Control | 1.98 | [-4.88, 8.85] | .563 | .967 | 0.009 | 0.19 |
| LR_Hipp | TE_age (Group+Age) | ADHD > Control | 2.34 | [-4.37, 9.05] | .485 | .938 | 0.012 | 0.23 |
| LR_Hipp | DE_BDI_adj (Group+BDI+Age+Sex) | ADHD > Control | 0.29 | [-8.63, 9.22] | .948 | .975 | 0.000 | 0.03 |
| LR_Hipp | DE_BDI_age (Group+BDI+Age) | ADHD > Control | 0.57 | [-8.06, 9.19] | .895 | .986 | 0.000 | 0.05 |
| LR_Occipital | TE_adj (Group+Age+Sex) | ADHD > Control | 0.02 | [-2.27, 2.30] | .987 | .996 | 0.000 | 0.01 |
| LR_Occipital | TE_age (Group+Age) | ADHD > Control | 0.43 | [-1.47, 2.33] | .637 | .938 | 0.015 | 0.22 |
| LR_Occipital | DE_BDI_adj (Group+BDI+Age+Sex) | ADHD < Control | -1.14 | [-3.45, 1.16] | .304 | .784 | 0.081 | -0.76 |
| LR_Occipital | DE_BDI_age (Group+BDI+Age) | ADHD < Control | -0.80 | [-3.20, 1.60] | .485 | .831 | 0.036 | -0.50 |
| LR_Pallidum | TE_adj (Group+Age+Sex) | ADHD < Control | -7.45 | [-25.60, 10.69] | .411 | .967 | 0.018 | -0.29 |
| LR_Pallidum | TE_age (Group+Age) | ADHD < Control | -6.30 | [-23.56, 10.97] | .465 | .938 | 0.014 | -0.24 |
| LR_Pallidum | DE_BDI_adj (Group+BDI+Age+Sex) | ADHD < Control | -8.85 | [-29.45, 11.75] | .390 | .784 | 0.020 | -0.34 |
| LR_Pallidum | DE_BDI_age (Group+BDI+Age) | ADHD < Control | -7.93 | [-28.28, 12.42] | .435 | .809 | 0.016 | -0.30 |
| LR_Putamen | TE_adj (Group+Age+Sex) | ADHD > Control | 1.24 | [-5.94, 8.42] | .728 | .967 | 0.004 | 0.12 |
| LR_Putamen | TE_age (Group+Age) | ADHD > Control | 1.27 | [-5.67, 8.21] | .713 | .938 | 0.004 | 0.12 |
| LR_Putamen | DE_BDI_adj (Group+BDI+Age+Sex) | ADHD > Control | 4.86 | [-2.83, 12.56] | .207 | .784 | 0.046 | 0.49 |
| LR_Putamen | DE_BDI_age (Group+BDI+Age) | ADHD > Control | 4.96 | [-2.31, 12.22] | .175 | .751 | 0.052 | 0.51 |
| L_Accumbens_area | TE_adj (Group+Age+Sex) | ADHD < Control | -6.77 | [-23.82, 10.28] | .427 | .967 | 0.016 | -0.26 |
| L_Accumbens_area | TE_age (Group+Age) | ADHD < Control | -6.52 | [-23.22, 10.17] | .434 | .938 | 0.015 | -0.25 |
| L_Accumbens_area | DE_BDI_adj (Group+BDI+Age+Sex) | ADHD < Control | -10.79 | [-30.73, 9.14] | .280 | .784 | 0.031 | -0.41 |
| L_Accumbens_area | DE_BDI_age (Group+BDI+Age) | ADHD < Control | -10.64 | [-30.25, 8.96] | .279 | .809 | 0.030 | -0.41 |
| L_Amygdala | TE_adj (Group+Age+Sex) | ADHD < Control | -2.56 | [-14.19, 9.07] | .659 | .967 | 0.005 | -0.17 |
| L_Amygdala | TE_age (Group+Age) | ADHD < Control | -1.97 | [-12.35, 8.41] | .703 | .938 | 0.004 | -0.13 |
| L_Amygdala | DE_BDI_adj (Group+BDI+Age+Sex) | ADHD < Control | -5.25 | [-17.64, 7.14] | .397 | .784 | 0.019 | -0.36 |
| L_Amygdala | DE_BDI_age (Group+BDI+Age) | ADHD < Control | -4.80 | [-16.76, 7.17] | .422 | .809 | 0.017 | -0.33 |
| L_AngularGyrus | TE_adj (Group+Age+Sex) | ADHD > Control | 0.11 | [-1.78, 2.00] | .909 | .996 | 0.000 | 0.04 |
| L_AngularGyrus | TE_age (Group+Age) | ADHD > Control | 0.01 | [-1.70, 1.73] | .989 | .990 | 0.000 | 0.00 |
| L_AngularGyrus | DE_BDI_adj (Group+BDI+Age+Sex) | ADHD > Control | 0.77 | [-1.44, 2.97] | .485 | .833 | 0.013 | 0.28 |
| L_AngularGyrus | DE_BDI_age (Group+BDI+Age) | ADHD > Control | 0.70 | [-1.35, 2.74] | .494 | .831 | 0.012 | 0.25 |
| L_CaudalAnteriorCingulate | TE_adj (Group+Age+Sex) | ADHD > Control | 1.98 | [-2.20, 6.16] | .343 | .967 | 0.023 | 0.30 |
| L_CaudalAnteriorCingulate | TE_age (Group+Age) | ADHD > Control | 1.71 | [-2.60, 6.02] | .427 | .938 | 0.016 | 0.26 |
| L_CaudalAnteriorCingulate | DE_BDI_adj (Group+BDI+Age+Sex) | ADHD > Control | 1.67 | [-3.73, 7.07] | .535 | .863 | 0.010 | 0.25 |
| L_CaudalAnteriorCingulate | DE_BDI_age (Group+BDI+Age) | ADHD > Control | 1.44 | [-4.18, 7.07] | .607 | .840 | 0.007 | 0.22 |
| L_CaudalMiddleFrontal | TE_adj (Group+Age+Sex) | ADHD > Control | 0.43 | [-2.00, 2.86] | .724 | .967 | 0.003 | 0.10 |
| L_CaudalMiddleFrontal | TE_age (Group+Age) | ADHD > Control | 0.63 | [-1.85, 3.10] | .612 | .938 | 0.007 | 0.15 |
| L_CaudalMiddleFrontal | DE_BDI_adj (Group+BDI+Age+Sex) | ADHD > Control | 1.39 | [-1.54, 4.31] | .343 | .784 | 0.024 | 0.34 |
| L_CaudalMiddleFrontal | DE_BDI_age (Group+BDI+Age) | ADHD > Control | 1.56 | [-1.34, 4.46] | .283 | .809 | 0.029 | 0.38 |
| L_Caudate | TE_adj (Group+Age+Sex) | ADHD < Control | -0.22 | [-9.83, 9.39] | .964 | .996 | 0.000 | -0.02 |
| L_Caudate | TE_age (Group+Age) | ADHD > Control | 0.68 | [-8.70, 10.06] | .884 | .962 | 0.001 | 0.05 |
| L_Caudate | DE_BDI_adj (Group+BDI+Age+Sex) | ADHD < Control | -0.82 | [-10.31, 8.67] | .862 | .975 | 0.001 | -0.06 |
| L_Caudate | DE_BDI_age (Group+BDI+Age) | ADHD < Control | -0.09 | [-9.70, 9.53] | .986 | .986 | 0.000 | -0.01 |
| L_Cuneus | TE_adj (Group+Age+Sex) | ADHD < Control | -1.13 | [-4.92, 2.66] | .549 | .967 | 0.009 | -0.22 |
| L_Cuneus | TE_age (Group+Age) | ADHD < Control | -1.00 | [-4.62, 2.63] | .580 | .938 | 0.008 | -0.20 |
| L_Cuneus | DE_BDI_adj (Group+BDI+Age+Sex) | ADHD < Control | -0.25 | [-4.23, 3.74] | .901 | .975 | 0.000 | -0.05 |
| L_Cuneus | DE_BDI_age (Group+BDI+Age) | ADHD < Control | -0.13 | [-3.96, 3.71] | .947 | .986 | 0.000 | -0.03 |
| L_DLPFC | TE_adj (Group+Age+Sex) | ADHD > Control | 0.45 | [-1.67, 2.57] | .666 | .967 | 0.007 | 0.16 |
| L_DLPFC | TE_age (Group+Age) | ADHD > Control | 0.48 | [-1.70, 2.67] | .653 | .938 | 0.008 | 0.17 |
| L_DLPFC | DE_BDI_adj (Group+BDI+Age+Sex) | ADHD > Control | 1.50 | [-0.81, 3.82] | .193 | .784 | 0.067 | 0.57 |
| L_DLPFC | DE_BDI_age (Group+BDI+Age) | ADHD > Control | 1.57 | [-0.71, 3.85] | .170 | .751 | 0.071 | 0.60 |
| L_Entorhinal | TE_adj (Group+Age+Sex) | ADHD < Control | -10.76 | [-22.20, 0.68] | .064 | .967 | 0.085 | -0.64 |
| L_Entorhinal | TE_age (Group+Age) | ADHD < Control | -10.77 | [-22.03, 0.49] | .060 | .938 | 0.085 | -0.64 |
| **L_Entorhinal** | DE_BDI_adj (Group+BDI+Age+Sex) | ADHD < Control | **-15.15** | **[-27.70, -2.59]** | **.019** | **.675** | **0.136** | **-0.91** |
| **L_Entorhinal** | DE_BDI_age (Group+BDI+Age) | ADHD < Control | **-15.21** | **[-27.47, -2.94]** | **.016** | **.600** | **0.139** | **-0.93** |
| L_Fusiform | TE_adj (Group+Age+Sex) | ADHD < Control | -3.71 | [-7.47, 0.04] | .053 | .967 | 0.093 | -0.69 |
| **L_Fusiform** | TE_age (Group+Age) | ADHD < Control | **-3.80** | **[-7.30, -0.30]** | **.034** | **.938** | **0.107** | **-0.72** |
| **L_Fusiform** | DE_BDI_adj (Group+BDI+Age+Sex) | ADHD < Control | **-4.23** | **[-8.14, -0.31]** | **.035** | **.675** | **0.112** | **-0.78** |
| **L_Fusiform** | DE_BDI_age (Group+BDI+Age) | ADHD < Control | **-4.30** | **[-7.97, -0.63]** | **.023** | **.600** | **0.126** | **-0.81** |
| L_Hipp | TE_adj (Group+Age+Sex) | ADHD > Control | 0.26 | [-6.52, 7.04] | .938 | .996 | 0.000 | 0.03 |
| L_Hipp | TE_age (Group+Age) | ADHD > Control | 1.44 | [-5.56, 8.44] | .679 | .938 | 0.004 | 0.13 |
| L_Hipp | DE_BDI_adj (Group+BDI+Age+Sex) | ADHD < Control | -1.25 | [-10.10, 7.60] | .776 | .943 | 0.002 | -0.12 |
| L_Hipp | DE_BDI_age (Group+BDI+Age) | ADHD < Control | -0.30 | [-9.34, 8.75] | .947 | .986 | 0.000 | -0.03 |
| L_InferiorParietal | TE_adj (Group+Age+Sex) | ADHD < Control | -0.17 | [-1.47, 1.13] | .793 | .983 | 0.002 | -0.09 |
| L_InferiorParietal | TE_age (Group+Age) | ADHD < Control | -0.19 | [-1.41, 1.03] | .755 | .953 | 0.002 | -0.10 |
| L_InferiorParietal | DE_BDI_adj (Group+BDI+Age+Sex) | ADHD > Control | 0.06 | [-1.40, 1.52] | .938 | .975 | 0.000 | 0.03 |
| L_InferiorParietal | DE_BDI_age (Group+BDI+Age) | ADHD > Control | 0.04 | [-1.34, 1.42] | .951 | .986 | 0.000 | 0.02 |
| L_InferiorTemporal | TE_adj (Group+Age+Sex) | ADHD < Control | -2.34 | [-6.74, 2.07] | .289 | .967 | 0.029 | -0.31 |
| L_InferiorTemporal | TE_age (Group+Age) | ADHD < Control | -2.23 | [-6.72, 2.26] | .322 | .938 | 0.025 | -0.30 |
| L_InferiorTemporal | DE_BDI_adj (Group+BDI+Age+Sex) | ADHD < Control | -4.70 | [-10.80, 1.40] | .127 | .784 | 0.062 | -0.64 |
| L_InferiorTemporal | DE_BDI_age (Group+BDI+Age) | ADHD < Control | -4.64 | [-10.60, 1.32] | .123 | .751 | 0.061 | -0.64 |
| L_Insula | TE_adj (Group+Age+Sex) | ADHD > Control | 0.91 | [-2.21, 4.04] | .558 | .967 | 0.009 | 0.19 |
| L_Insula | TE_age (Group+Age) | ADHD > Control | 1.20 | [-2.06, 4.46] | .461 | .938 | 0.014 | 0.25 |
| L_Insula | DE_BDI_adj (Group+BDI+Age+Sex) | ADHD > Control | 1.66 | [-1.87, 5.19] | .347 | .784 | 0.023 | 0.34 |
| L_Insula | DE_BDI_age (Group+BDI+Age) | ADHD > Control | 1.90 | [-1.84, 5.65] | .310 | .809 | 0.026 | 0.39 |
| L_IsthmusCingulate | TE_adj (Group+Age+Sex) | ADHD < Control | -0.65 | [-5.75, 4.45] | .798 | .983 | 0.002 | -0.09 |
| L_IsthmusCingulate | TE_age (Group+Age) | ADHD < Control | -0.43 | [-5.09, 4.23] | .852 | .962 | 0.001 | -0.06 |
| L_IsthmusCingulate | DE_BDI_adj (Group+BDI+Age+Sex) | ADHD > Control | 0.25 | [-5.55, 6.04] | .932 | .975 | 0.000 | 0.04 |
| L_IsthmusCingulate | DE_BDI_age (Group+BDI+Age) | ADHD > Control | 0.44 | [-4.93, 5.80] | .870 | .986 | 0.001 | 0.06 |
| L_LateralOccipital | TE_adj (Group+Age+Sex) | ADHD < Control | -0.95 | [-2.70, 0.79] | .276 | .967 | 0.030 | -0.34 |
| L_LateralOccipital | TE_age (Group+Age) | ADHD < Control | -1.06 | [-2.82, 0.70] | .232 | .938 | 0.036 | -0.38 |
| L_LateralOccipital | DE_BDI_adj (Group+BDI+Age+Sex) | ADHD < Control | -1.23 | [-3.20, 0.74] | .214 | .784 | 0.040 | -0.44 |
| L_LateralOccipital | DE_BDI_age (Group+BDI+Age) | ADHD < Control | -1.32 | [-3.27, 0.64] | .181 | .751 | 0.046 | -0.47 |
| L_LateralOrbitofrontal | TE_adj (Group+Age+Sex) | ADHD < Control | -2.83 | [-8.31, 2.65] | .303 | .967 | 0.027 | -0.35 |
| L_LateralOrbitofrontal | TE_age (Group+Age) | ADHD < Control | -2.57 | [-7.77, 2.63] | .324 | .938 | 0.024 | -0.32 |
| L_LateralOrbitofrontal | DE_BDI_adj (Group+BDI+Age+Sex) | ADHD < Control | -4.60 | [-10.94, 1.74] | .150 | .784 | 0.054 | -0.57 |
| L_LateralOrbitofrontal | DE_BDI_age (Group+BDI+Age) | ADHD < Control | -4.41 | [-10.58, 1.77] | .157 | .751 | 0.051 | -0.55 |
| L_Lingual | TE_adj (Group+Age+Sex) | ADHD < Control | -1.12 | [-5.37, 3.13] | .598 | .967 | 0.007 | -0.17 |
| L_Lingual | TE_age (Group+Age) | ADHD < Control | -1.06 | [-5.36, 3.25] | .623 | .938 | 0.006 | -0.16 |
| L_Lingual | DE_BDI_adj (Group+BDI+Age+Sex) | ADHD < Control | -1.99 | [-6.83, 2.85] | .411 | .784 | 0.018 | -0.29 |
| L_Lingual | DE_BDI_age (Group+BDI+Age) | ADHD < Control | -1.95 | [-6.71, 2.81] | .413 | .809 | 0.017 | -0.29 |
| L_MedialOrbitofrontal | TE_adj (Group+Age+Sex) | ADHD < Control | -4.27 | [-10.40, 1.86] | .167 | .967 | 0.048 | -0.47 |
| L_MedialOrbitofrontal | TE_age (Group+Age) | ADHD < Control | -3.95 | [-9.75, 1.86] | .177 | .938 | 0.045 | -0.44 |
| **L_MedialOrbitofrontal** | DE_BDI_adj (Group+BDI+Age+Sex) | ADHD < Control | **-7.18** | **[-14.18, -0.17]** | **.045** | **.675** | **0.102** | **-0.82** |
| **L_MedialOrbitofrontal** | DE_BDI_age (Group+BDI+Age) | ADHD < Control | **-6.94** | **[-13.72, -0.16]** | **.045** | **.643** | **0.099** | **-0.80** |
| L_MiddleTemporal | TE_adj (Group+Age+Sex) | ADHD < Control | -0.48 | [-2.39, 1.43] | .612 | .967 | 0.007 | -0.18 |
| L_MiddleTemporal | TE_age (Group+Age) | ADHD < Control | -0.39 | [-2.17, 1.40] | .665 | .938 | 0.005 | -0.14 |
| L_MiddleTemporal | DE_BDI_adj (Group+BDI+Age+Sex) | ADHD < Control | -1.40 | [-3.46, 0.66] | .178 | .784 | 0.047 | -0.54 |
| L_MiddleTemporal | DE_BDI_age (Group+BDI+Age) | ADHD < Control | -1.33 | [-3.35, 0.69] | .190 | .752 | 0.044 | -0.51 |
| L_Occipital | TE_adj (Group+Age+Sex) | ADHD < Control | -1.10 | [-3.41, 1.22] | .343 | .967 | 0.023 | -0.31 |
| L_Occipital | TE_age (Group+Age) | ADHD < Control | -1.09 | [-3.42, 1.25] | .353 | .938 | 0.022 | -0.31 |
| L_Occipital | DE_BDI_adj (Group+BDI+Age+Sex) | ADHD < Control | -1.32 | [-3.83, 1.19] | .295 | .784 | 0.029 | -0.37 |
| L_Occipital | DE_BDI_age (Group+BDI+Age) | ADHD < Control | -1.31 | [-3.77, 1.15] | .287 | .809 | 0.029 | -0.37 |
| L_Pallidum | TE_adj (Group+Age+Sex) | ADHD < Control | -8.71 | [-28.99, 11.57] | .390 | .967 | 0.019 | -0.29 |
| L_Pallidum | TE_age (Group+Age) | ADHD < Control | -6.87 | [-26.75, 13.01] | .489 | .938 | 0.012 | -0.22 |
| L_Pallidum | DE_BDI_adj (Group+BDI+Age+Sex) | ADHD < Control | -9.69 | [-32.72, 13.35] | .400 | .784 | 0.019 | -0.31 |
| L_Pallidum | DE_BDI_age (Group+BDI+Age) | ADHD < Control | -8.18 | [-31.22, 14.85] | .477 | .831 | 0.013 | -0.26 |
| L_Parahippocampal | TE_adj (Group+Age+Sex) | ADHD < Control | -0.02 | [-8.46, 8.41] | .996 | .996 | 0.000 | -0.00 |
| L_Parahippocampal | TE_age (Group+Age) | ADHD < Control | -0.31 | [-8.50, 7.87] | .939 | .963 | 0.000 | -0.03 |
| L_Parahippocampal | DE_BDI_adj (Group+BDI+Age+Sex) | ADHD < Control | -1.11 | [-10.82, 8.60] | .818 | .967 | 0.001 | -0.09 |
| L_Parahippocampal | DE_BDI_age (Group+BDI+Age) | ADHD < Control | -1.36 | [-10.85, 8.12] | .773 | .918 | 0.002 | -0.11 |
| L_Pericalcarine | TE_adj (Group+Age+Sex) | ADHD < Control | -1.50 | [-4.89, 1.88] | .375 | .967 | 0.020 | -0.29 |
| L_Pericalcarine | TE_age (Group+Age) | ADHD < Control | -1.31 | [-4.85, 2.24] | .461 | .938 | 0.014 | -0.25 |
| L_Pericalcarine | DE_BDI_adj (Group+BDI+Age+Sex) | ADHD < Control | -3.23 | [-6.87, 0.42] | .081 | .675 | 0.078 | -0.64 |
| L_Pericalcarine | DE_BDI_age (Group+BDI+Age) | ADHD < Control | -3.09 | [-6.77, 0.59] | .098 | .721 | 0.069 | -0.62 |
| L_Postcentral | TE_adj (Group+Age+Sex) | ADHD > Control | 0.32 | [-1.27, 1.90] | .687 | .967 | 0.004 | 0.12 |
| L_Postcentral | TE_age (Group+Age) | ADHD > Control | 0.56 | [-1.11, 2.24] | .500 | .938 | 0.011 | 0.20 |
| L_Postcentral | DE_BDI_adj (Group+BDI+Age+Sex) | ADHD > Control | 1.20 | [-0.59, 2.99] | .184 | .784 | 0.046 | 0.46 |
| L_Postcentral | DE_BDI_age (Group+BDI+Age) | ADHD > Control | 1.41 | [-0.42, 3.25] | .127 | .751 | 0.059 | 0.52 |
| **L_PosteriorCingulate** | TE_adj (Group+Age+Sex) | ADHD < Control | **-2.84** | **[-5.45, -0.24]** | **.033** | **.967** | **0.111** | **-0.78** |
| **L_PosteriorCingulate** | TE_age (Group+Age) | ADHD < Control | **-2.58** | **[-5.13, -0.02]** | **.048** | **.938** | **0.094** | **-0.70** |
| L_PosteriorCingulate | DE_BDI_adj (Group+BDI+Age+Sex) | ADHD < Control | -1.97 | [-4.86, 0.93] | .177 | .784 | 0.047 | -0.55 |
| L_PosteriorCingulate | DE_BDI_age (Group+BDI+Age) | ADHD < Control | -1.74 | [-4.60, 1.13] | .228 | .809 | 0.037 | -0.48 |
| L_Precentral | TE_adj (Group+Age+Sex) | ADHD > Control | 1.25 | [-0.62, 3.13] | .184 | .967 | 0.045 | 0.42 |
| L_Precentral | TE_age (Group+Age) | ADHD > Control | 1.52 | [-0.43, 3.46] | .123 | .938 | 0.058 | 0.49 |
| L_Precentral | DE_BDI_adj (Group+BDI+Age+Sex) | ADHD > Control | 1.88 | [-0.27, 4.03] | .085 | .675 | 0.076 | 0.63 |
| L_Precentral | DE_BDI_age (Group+BDI+Age) | ADHD > Control | 2.10 | [-0.12, 4.32] | .063 | .643 | 0.086 | 0.68 |
| L_Precuneus | TE_adj (Group+Age+Sex) | ADHD < Control | -0.63 | [-2.82, 1.57] | .566 | .967 | 0.009 | -0.19 |
| L_Precuneus | TE_age (Group+Age) | ADHD < Control | -0.50 | [-2.66, 1.66] | .643 | .938 | 0.005 | -0.15 |
| L_Precuneus | DE_BDI_adj (Group+BDI+Age+Sex) | ADHD > Control | 0.27 | [-2.10, 2.63] | .820 | .967 | 0.001 | 0.08 |
| L_Precuneus | DE_BDI_age (Group+BDI+Age) | ADHD > Control | 0.38 | [-1.91, 2.67] | .736 | .918 | 0.003 | 0.12 |
| L_Putamen | TE_adj (Group+Age+Sex) | ADHD > Control | 4.03 | [-5.65, 13.72] | .405 | .967 | 0.018 | 0.27 |
| L_Putamen | TE_age (Group+Age) | ADHD > Control | 4.17 | [-4.80, 13.15] | .353 | .938 | 0.022 | 0.29 |
| L_Putamen | DE_BDI_adj (Group+BDI+Age+Sex) | ADHD > Control | 4.53 | [-6.31, 15.38] | .403 | .784 | 0.018 | 0.30 |
| L_Putamen | DE_BDI_age (Group+BDI+Age) | ADHD > Control | 4.66 | [-5.62, 14.93] | .365 | .809 | 0.021 | 0.32 |
| L_RostralAnteriorCingulate | TE_adj (Group+Age+Sex) | ADHD > Control | 0.75 | [-3.45, 4.96] | .719 | .967 | 0.003 | 0.12 |
| L_RostralAnteriorCingulate | TE_age (Group+Age) | ADHD > Control | 0.98 | [-3.03, 5.00] | .623 | .938 | 0.006 | 0.16 |
| L_RostralAnteriorCingulate | DE_BDI_adj (Group+BDI+Age+Sex) | ADHD > Control | 1.25 | [-4.06, 6.55] | .637 | .911 | 0.006 | 0.19 |
| L_RostralAnteriorCingulate | DE_BDI_age (Group+BDI+Age) | ADHD > Control | 1.44 | [-3.62, 6.50] | .568 | .840 | 0.008 | 0.23 |
| L_RostralMiddleFrontal | TE_adj (Group+Age+Sex) | ADHD > Control | 0.13 | [-1.36, 1.63] | .856 | .983 | 0.001 | 0.06 |
| L_RostralMiddleFrontal | TE_age (Group+Age) | ADHD > Control | 0.29 | [-1.20, 1.78] | .698 | .938 | 0.004 | 0.12 |
| L_RostralMiddleFrontal | DE_BDI_adj (Group+BDI+Age+Sex) | ADHD > Control | 0.26 | [-1.26, 1.78] | .730 | .923 | 0.003 | 0.11 |
| L_RostralMiddleFrontal | DE_BDI_age (Group+BDI+Age) | ADHD > Control | 0.39 | [-1.14, 1.92] | .610 | .840 | 0.007 | 0.16 |
| L_SuperiorFrontal | TE_adj (Group+Age+Sex) | ADHD > Control | 0.03 | [-2.25, 2.31] | .977 | .996 | 0.000 | 0.01 |
| L_SuperiorFrontal | TE_age (Group+Age) | ADHD > Control | 0.15 | [-2.10, 2.40] | .894 | .962 | 0.000 | 0.04 |
| L_SuperiorFrontal | DE_BDI_adj (Group+BDI+Age+Sex) | ADHD > Control | 0.50 | [-2.09, 3.09] | .697 | .923 | 0.004 | 0.14 |
| L_SuperiorFrontal | DE_BDI_age (Group+BDI+Age) | ADHD > Control | 0.58 | [-1.95, 3.12] | .644 | .863 | 0.006 | 0.17 |
| L_SuperiorParietal | TE_adj (Group+Age+Sex) | ADHD > Control | 0.30 | [-1.47, 2.07] | .737 | .967 | 0.003 | 0.11 |
| L_SuperiorParietal | TE_age (Group+Age) | ADHD > Control | 0.24 | [-1.34, 1.81] | .764 | .953 | 0.002 | 0.09 |
| L_SuperiorParietal | DE_BDI_adj (Group+BDI+Age+Sex) | ADHD > Control | 0.98 | [-0.85, 2.81] | .286 | .784 | 0.030 | 0.37 |
| L_SuperiorParietal | DE_BDI_age (Group+BDI+Age) | ADHD > Control | 0.94 | [-0.72, 2.59] | .260 | .809 | 0.032 | 0.36 |
| L_SuperiorTemporal | TE_adj (Group+Age+Sex) | ADHD > Control | 0.47 | [-2.22, 3.16] | .726 | .967 | 0.003 | 0.13 |
| L_SuperiorTemporal | TE_age (Group+Age) | ADHD > Control | 0.65 | [-1.88, 3.19] | .605 | .938 | 0.007 | 0.18 |
| L_SuperiorTemporal | DE_BDI_adj (Group+BDI+Age+Sex) | ADHD > Control | 0.11 | [-2.67, 2.89] | .937 | .975 | 0.000 | 0.03 |
| L_SuperiorTemporal | DE_BDI_age (Group+BDI+Age) | ADHD > Control | 0.26 | [-2.50, 3.01] | .851 | .986 | 0.001 | 0.07 |
| L_Thalamus | TE_adj (Group+Age+Sex) | ADHD > Control | 1.36 | [-3.01, 5.74] | .532 | .967 | 0.010 | 0.21 |
| L_Thalamus | TE_age (Group+Age) | ADHD > Control | 1.87 | [-2.52, 6.26] | .394 | .938 | 0.018 | 0.28 |
| L_Thalamus | DE_BDI_adj (Group+BDI+Age+Sex) | ADHD > Control | 2.26 | [-2.97, 7.50] | .387 | .784 | 0.020 | 0.34 |
| L_Thalamus | DE_BDI_age (Group+BDI+Age) | ADHD > Control | 2.69 | [-2.61, 7.99] | .311 | .809 | 0.026 | 0.40 |
| L_TransverseTemporal | TE_adj (Group+Age+Sex) | ADHD < Control | -1.64 | [-5.10, 1.81] | .342 | .967 | 0.023 | -0.28 |
| L_TransverseTemporal | TE_age (Group+Age) | ADHD < Control | -1.20 | [-4.79, 2.40] | .505 | .938 | 0.011 | -0.20 |
| L_TransverseTemporal | DE_BDI_adj (Group+BDI+Age+Sex) | ADHD < Control | -0.69 | [-5.39, 4.01] | .768 | .943 | 0.002 | -0.12 |
| L_TransverseTemporal | DE_BDI_age (Group+BDI+Age) | ADHD < Control | -0.31 | [-5.17, 4.55] | .898 | .986 | 0.000 | -0.05 |
| R_Accumbens_area | TE_adj (Group+Age+Sex) | ADHD < Control | -4.09 | [-19.09, 10.92] | .585 | .967 | 0.008 | -0.18 |
| R_Accumbens_area | TE_age (Group+Age) | ADHD < Control | -3.66 | [-18.68, 11.35] | .625 | .938 | 0.006 | -0.16 |
| R_Accumbens_area | DE_BDI_adj (Group+BDI+Age+Sex) | ADHD < Control | -7.51 | [-25.93, 10.90] | .414 | .784 | 0.018 | -0.33 |
| R_Accumbens_area | DE_BDI_age (Group+BDI+Age) | ADHD < Control | -7.21 | [-25.75, 11.33] | .436 | .809 | 0.016 | -0.32 |
| R_Amygdala | TE_adj (Group+Age+Sex) | ADHD > Control | 2.85 | [-8.65, 14.34] | .619 | .967 | 0.006 | 0.15 |
| R_Amygdala | TE_age (Group+Age) | ADHD > Control | 3.35 | [-8.88, 15.58] | .583 | .938 | 0.008 | 0.18 |
| R_Amygdala | DE_BDI_adj (Group+BDI+Age+Sex) | ADHD < Control | -2.51 | [-17.46, 12.44] | .736 | .923 | 0.003 | -0.14 |
| R_Amygdala | DE_BDI_age (Group+BDI+Age) | ADHD < Control | -2.16 | [-17.59, 13.26] | .778 | .918 | 0.002 | -0.12 |
| R_AngularGyrus | TE_adj (Group+Age+Sex) | ADHD < Control | -0.19 | [-2.22, 1.84] | .852 | .983 | 0.001 | -0.06 |
| R_AngularGyrus | TE_age (Group+Age) | ADHD < Control | -0.10 | [-2.08, 1.88] | .921 | .962 | 0.000 | -0.03 |
| R_AngularGyrus | DE_BDI_adj (Group+BDI+Age+Sex) | ADHD > Control | 0.54 | [-1.72, 2.80] | .630 | .911 | 0.006 | 0.19 |
| R_AngularGyrus | DE_BDI_age (Group+BDI+Age) | ADHD > Control | 0.63 | [-1.58, 2.83] | .570 | .840 | 0.008 | 0.22 |
| R_CaudalAnteriorCingulate | TE_adj (Group+Age+Sex) | ADHD < Control | -3.64 | [-9.12, 1.85] | .188 | .967 | 0.044 | -0.37 |
| R_CaudalAnteriorCingulate | TE_age (Group+Age) | ADHD < Control | -3.93 | [-9.78, 1.93] | .183 | .938 | 0.044 | -0.41 |
| R_CaudalAnteriorCingulate | DE_BDI_adj (Group+BDI+Age+Sex) | ADHD < Control | -4.42 | [-11.87, 3.04] | .238 | .784 | 0.037 | -0.45 |
| R_CaudalAnteriorCingulate | DE_BDI_age (Group+BDI+Age) | ADHD < Control | -4.67 | [-12.50, 3.17] | .236 | .809 | 0.036 | -0.48 |
| R_CaudalMiddleFrontal | TE_adj (Group+Age+Sex) | ADHD > Control | 0.45 | [-2.06, 2.96] | .720 | .967 | 0.003 | 0.12 |
| R_CaudalMiddleFrontal | TE_age (Group+Age) | ADHD > Control | 0.70 | [-1.76, 3.16] | .567 | .938 | 0.008 | 0.18 |
| R_CaudalMiddleFrontal | DE_BDI_adj (Group+BDI+Age+Sex) | ADHD > Control | 1.11 | [-1.64, 3.86] | .420 | .784 | 0.017 | 0.29 |
| R_CaudalMiddleFrontal | DE_BDI_age (Group+BDI+Age) | ADHD > Control | 1.32 | [-1.41, 4.06] | .333 | .809 | 0.024 | 0.35 |
| R_Caudate | TE_adj (Group+Age+Sex) | ADHD > Control | 2.95 | [-5.88, 11.79] | .503 | .967 | 0.012 | 0.22 |
| R_Caudate | TE_age (Group+Age) | ADHD > Control | 3.75 | [-4.72, 12.21] | .377 | .938 | 0.020 | 0.28 |
| R_Caudate | DE_BDI_adj (Group+BDI+Age+Sex) | ADHD > Control | 3.89 | [-5.09, 12.88] | .386 | .784 | 0.020 | 0.29 |
| R_Caudate | DE_BDI_age (Group+BDI+Age) | ADHD > Control | 4.56 | [-4.40, 13.51] | .310 | .809 | 0.026 | 0.34 |
| R_Cuneus | TE_adj (Group+Age+Sex) | ADHD < Control | -1.36 | [-7.60, 4.88] | .661 | .967 | 0.005 | -0.20 |
| R_Cuneus | TE_age (Group+Age) | ADHD < Control | -1.13 | [-6.87, 4.62] | .694 | .938 | 0.004 | -0.17 |
| R_Cuneus | DE_BDI_adj (Group+BDI+Age+Sex) | ADHD < Control | -0.32 | [-6.48, 5.84] | .916 | .975 | 0.000 | -0.05 |
| R_Cuneus | DE_BDI_age (Group+BDI+Age) | ADHD < Control | -0.12 | [-5.84, 5.60] | .967 | .986 | 0.000 | -0.02 |
| R_DLPFC | TE_adj (Group+Age+Sex) | ADHD < Control | -0.35 | [-2.56, 1.87] | .747 | .967 | 0.005 | -0.13 |
| R_DLPFC | TE_age (Group+Age) | ADHD < Control | -0.30 | [-2.64, 2.03] | .789 | .953 | 0.003 | -0.12 |
| R_DLPFC | DE_BDI_adj (Group+BDI+Age+Sex) | ADHD > Control | 0.92 | [-1.75, 3.58] | .481 | .833 | 0.026 | 0.38 |
| R_DLPFC | DE_BDI_age (Group+BDI+Age) | ADHD > Control | 0.97 | [-1.61, 3.56] | .440 | .809 | 0.030 | 0.41 |
| R_Entorhinal | TE_adj (Group+Age+Sex) | ADHD < Control | -1.81 | [-9.37, 5.75] | .632 | .967 | 0.006 | -0.13 |
| R_Entorhinal | TE_age (Group+Age) | ADHD < Control | -1.95 | [-10.04, 6.15] | .630 | .938 | 0.006 | -0.15 |
| R_Entorhinal | DE_BDI_adj (Group+BDI+Age+Sex) | ADHD < Control | -4.71 | [-15.48, 6.06] | .381 | .784 | 0.020 | -0.35 |
| R_Entorhinal | DE_BDI_age (Group+BDI+Age) | ADHD < Control | -4.86 | [-15.95, 6.23] | .381 | .809 | 0.020 | -0.37 |
| **R_Fusiform** | TE_adj (Group+Age+Sex) | ADHD < Control | **-4.54** | **[-8.65, -0.43]** | **.031** | **.967** | **0.114** | **-0.72** |
| **R_Fusiform** | TE_age (Group+Age) | ADHD < Control | **-4.29** | **[-8.36, -0.21]** | **.040** | **.938** | **0.102** | **-0.68** |
| **R_Fusiform** | DE_BDI_adj (Group+BDI+Age+Sex) | ADHD < Control | **-5.63** | **[-9.94, -1.32]** | **.012** | **.675** | **0.155** | **-0.89** |
| **R_Fusiform** | DE_BDI_age (Group+BDI+Age) | ADHD < Control | **-5.43** | **[-9.55, -1.31]** | **.011** | **.600** | **0.154** | **-0.86** |
| R_Hipp | TE_adj (Group+Age+Sex) | ADHD > Control | 3.70 | [-4.13, 11.54] | .345 | .967 | 0.023 | 0.31 |
| R_Hipp | TE_age (Group+Age) | ADHD > Control | 3.38 | [-4.26, 11.01] | .377 | .938 | 0.020 | 0.29 |
| R_Hipp | DE_BDI_adj (Group+BDI+Age+Sex) | ADHD > Control | 2.02 | [-8.02, 12.06] | .686 | .923 | 0.004 | 0.17 |
| R_Hipp | DE_BDI_age (Group+BDI+Age) | ADHD > Control | 1.73 | [-8.10, 11.56] | .724 | .918 | 0.003 | 0.15 |
| R_InferiorParietal | TE_adj (Group+Age+Sex) | ADHD > Control | 0.01 | [-1.66, 1.67] | .995 | .996 | 0.000 | 0.00 |
| R_InferiorParietal | TE_age (Group+Age) | ADHD > Control | 0.09 | [-1.53, 1.71] | .910 | .962 | 0.000 | 0.04 |
| R_InferiorParietal | DE_BDI_adj (Group+BDI+Age+Sex) | ADHD > Control | 0.43 | [-1.34, 2.19] | .627 | .911 | 0.006 | 0.18 |
| R_InferiorParietal | DE_BDI_age (Group+BDI+Age) | ADHD > Control | 0.50 | [-1.22, 2.23] | .559 | .840 | 0.009 | 0.22 |
| R_InferiorTemporal | TE_adj (Group+Age+Sex) | ADHD < Control | -3.52 | [-8.44, 1.40] | .156 | .967 | 0.051 | -0.46 |
| R_InferiorTemporal | TE_age (Group+Age) | ADHD < Control | -3.37 | [-8.18, 1.44] | .164 | .938 | 0.048 | -0.44 |
| R_InferiorTemporal | DE_BDI_adj (Group+BDI+Age+Sex) | ADHD < Control | -5.45 | [-11.24, 0.34] | .064 | .675 | 0.087 | -0.72 |
| R_InferiorTemporal | DE_BDI_age (Group+BDI+Age) | ADHD < Control | -5.35 | [-10.98, 0.27] | .061 | .643 | 0.087 | -0.71 |
| R_Insula | TE_adj (Group+Age+Sex) | ADHD < Control | -0.10 | [-2.84, 2.63] | .940 | .996 | 0.000 | -0.03 |
| R_Insula | TE_age (Group+Age) | ADHD > Control | 0.17 | [-2.52, 2.85] | .902 | .962 | 0.000 | 0.04 |
| R_Insula | DE_BDI_adj (Group+BDI+Age+Sex) | ADHD > Control | 0.65 | [-2.24, 3.54] | .651 | .911 | 0.005 | 0.17 |
| R_Insula | DE_BDI_age (Group+BDI+Age) | ADHD > Control | 0.88 | [-1.97, 3.73] | .535 | .840 | 0.010 | 0.23 |
| R_IsthmusCingulate | TE_adj (Group+Age+Sex) | ADHD < Control | -0.74 | [-8.67, 7.19] | .851 | .983 | 0.001 | -0.07 |
| R_IsthmusCingulate | TE_age (Group+Age) | ADHD > Control | 0.05 | [-7.80, 7.90] | .990 | .990 | 0.000 | 0.00 |
| R_IsthmusCingulate | DE_BDI_adj (Group+BDI+Age+Sex) | ADHD > Control | 1.69 | [-7.22, 10.60] | .703 | .923 | 0.004 | 0.16 |
| R_IsthmusCingulate | DE_BDI_age (Group+BDI+Age) | ADHD > Control | 2.37 | [-6.50, 11.24] | .592 | .840 | 0.007 | 0.22 |
| R_LateralOccipital | TE_adj (Group+Age+Sex) | ADHD < Control | -1.44 | [-3.65, 0.76] | .193 | .967 | 0.043 | -0.46 |
| R_LateralOccipital | TE_age (Group+Age) | ADHD < Control | -1.44 | [-3.60, 0.72] | .186 | .938 | 0.043 | -0.47 |
| R_LateralOccipital | DE_BDI_adj (Group+BDI+Age+Sex) | ADHD < Control | -1.67 | [-4.06, 0.71] | .164 | .784 | 0.050 | -0.53 |
| R_LateralOccipital | DE_BDI_age (Group+BDI+Age) | ADHD < Control | -1.67 | [-3.98, 0.63] | .151 | .751 | 0.052 | -0.54 |
| R_LateralOrbitofrontal | TE_adj (Group+Age+Sex) | ADHD < Control | -3.40 | [-8.50, 1.70] | .185 | .967 | 0.045 | -0.45 |
| R_LateralOrbitofrontal | TE_age (Group+Age) | ADHD < Control | -3.12 | [-7.92, 1.68] | .197 | .938 | 0.041 | -0.41 |
| R_LateralOrbitofrontal | DE_BDI_adj (Group+BDI+Age+Sex) | ADHD < Control | -5.39 | [-11.16, 0.39] | .067 | .675 | 0.086 | -0.73 |
| R_LateralOrbitofrontal | DE_BDI_age (Group+BDI+Age) | ADHD < Control | -5.18 | [-10.74, 0.38] | .067 | .643 | 0.083 | -0.70 |
| R_Lingual | TE_adj (Group+Age+Sex) | ADHD < Control | -2.62 | [-7.82, 2.57] | .313 | .967 | 0.026 | -0.37 |
| R_Lingual | TE_age (Group+Age) | ADHD < Control | -2.45 | [-7.58, 2.67] | .339 | .938 | 0.023 | -0.35 |
| R_Lingual | DE_BDI_adj (Group+BDI+Age+Sex) | ADHD < Control | -3.92 | [-9.19, 1.36] | .141 | .784 | 0.056 | -0.56 |
| R_Lingual | DE_BDI_age (Group+BDI+Age) | ADHD < Control | -3.79 | [-8.89, 1.30] | .140 | .751 | 0.055 | -0.55 |
| R_MedialOrbitofrontal | TE_adj (Group+Age+Sex) | ADHD < Control | -4.53 | [-11.67, 2.61] | .207 | .967 | 0.041 | -0.41 |
| R_MedialOrbitofrontal | TE_age (Group+Age) | ADHD < Control | -4.53 | [-11.49, 2.42] | .195 | .938 | 0.042 | -0.41 |
| R_MedialOrbitofrontal | DE_BDI_adj (Group+BDI+Age+Sex) | ADHD < Control | -8.09 | [-16.56, 0.38] | .061 | .675 | 0.090 | -0.76 |
| R_MedialOrbitofrontal | DE_BDI_age (Group+BDI+Age) | ADHD < Control | -8.14 | [-16.43, 0.15] | .054 | .643 | 0.092 | -0.77 |
| R_MiddleTemporal | TE_adj (Group+Age+Sex) | ADHD < Control | -0.38 | [-1.98, 1.22] | .635 | .967 | 0.006 | -0.17 |
| R_MiddleTemporal | TE_age (Group+Age) | ADHD < Control | -0.20 | [-1.66, 1.26] | .784 | .953 | 0.002 | -0.09 |
| R_MiddleTemporal | DE_BDI_adj (Group+BDI+Age+Sex) | ADHD < Control | -0.84 | [-2.64, 0.97] | .354 | .784 | 0.023 | -0.38 |
| R_MiddleTemporal | DE_BDI_age (Group+BDI+Age) | ADHD < Control | -0.69 | [-2.47, 1.08] | .434 | .809 | 0.016 | -0.31 |
| R_Occipital | TE_adj (Group+Age+Sex) | ADHD < Control | -0.21 | [-2.76, 2.35] | .871 | .983 | 0.001 | -0.06 |
| R_Occipital | TE_age (Group+Age) | ADHD < Control | -0.14 | [-2.65, 2.36] | .908 | .962 | 0.000 | -0.04 |
| R_Occipital | DE_BDI_adj (Group+BDI+Age+Sex) | ADHD < Control | -0.64 | [-3.55, 2.27] | .657 | .911 | 0.006 | -0.18 |
| R_Occipital | DE_BDI_age (Group+BDI+Age) | ADHD < Control | -0.60 | [-3.38, 2.19] | .667 | .878 | 0.005 | -0.17 |
| R_Pallidum | TE_adj (Group+Age+Sex) | ADHD > Control | 1.64 | [-18.07, 21.34] | .867 | .983 | 0.001 | 0.05 |
| R_Pallidum | TE_age (Group+Age) | ADHD > Control | 3.06 | [-16.23, 22.35] | .750 | .953 | 0.003 | 0.10 |
| R_Pallidum | DE_BDI_adj (Group+BDI+Age+Sex) | ADHD < Control | -0.16 | [-22.92, 22.61] | .989 | .989 | 0.000 | -0.01 |
| R_Pallidum | DE_BDI_age (Group+BDI+Age) | ADHD > Control | 0.99 | [-21.68, 23.66] | .930 | .986 | 0.000 | 0.03 |
| R_Parahippocampal | TE_adj (Group+Age+Sex) | ADHD < Control | -5.13 | [-15.97, 5.71] | .345 | .967 | 0.023 | -0.30 |
| R_Parahippocampal | TE_age (Group+Age) | ADHD < Control | -6.09 | [-17.20, 5.02] | .275 | .938 | 0.030 | -0.36 |
| R_Parahippocampal | DE_BDI_adj (Group+BDI+Age+Sex) | ADHD < Control | -5.24 | [-18.53, 8.05] | .430 | .784 | 0.016 | -0.30 |
| R_Parahippocampal | DE_BDI_age (Group+BDI+Age) | ADHD < Control | -6.03 | [-19.91, 7.84] | .384 | .809 | 0.019 | -0.35 |
| R_Pericalcarine | TE_adj (Group+Age+Sex) | ADHD < Control | -0.87 | [-5.21, 3.46] | .686 | .967 | 0.004 | -0.15 |
| R_Pericalcarine | TE_age (Group+Age) | ADHD < Control | -0.54 | [-4.97, 3.89] | .808 | .953 | 0.001 | -0.09 |
| R_Pericalcarine | DE_BDI_adj (Group+BDI+Age+Sex) | ADHD < Control | -1.56 | [-6.38, 3.26] | .517 | .863 | 0.011 | -0.26 |
| R_Pericalcarine | DE_BDI_age (Group+BDI+Age) | ADHD < Control | -1.29 | [-6.16, 3.58] | .595 | .840 | 0.007 | -0.22 |
| R_Postcentral | TE_adj (Group+Age+Sex) | ADHD < Control | -0.42 | [-2.20, 1.35] | .632 | .967 | 0.006 | -0.16 |
| R_Postcentral | TE_age (Group+Age) | ADHD < Control | -0.22 | [-1.96, 1.52] | .799 | .953 | 0.002 | -0.08 |
| R_Postcentral | DE_BDI_adj (Group+BDI+Age+Sex) | ADHD > Control | 0.13 | [-1.89, 2.14] | .899 | .975 | 0.000 | 0.05 |
| R_Postcentral | DE_BDI_age (Group+BDI+Age) | ADHD > Control | 0.30 | [-1.68, 2.28] | .759 | .918 | 0.002 | 0.11 |
| R_PosteriorCingulate | TE_adj (Group+Age+Sex) | ADHD < Control | -0.89 | [-3.45, 1.66] | .483 | .967 | 0.013 | -0.23 |
| R_PosteriorCingulate | TE_age (Group+Age) | ADHD < Control | -0.77 | [-3.21, 1.68] | .530 | .938 | 0.010 | -0.20 |
| R_PosteriorCingulate | DE_BDI_adj (Group+BDI+Age+Sex) | ADHD < Control | -0.07 | [-3.01, 2.87] | .963 | .975 | 0.000 | -0.02 |
| R_PosteriorCingulate | DE_BDI_age (Group+BDI+Age) | ADHD > Control | 0.05 | [-2.80, 2.89] | .973 | .986 | 0.000 | 0.01 |
| R_Precentral | TE_adj (Group+Age+Sex) | ADHD > Control | 0.29 | [-1.20, 1.77] | .698 | .967 | 0.004 | 0.12 |
| R_Precentral | TE_age (Group+Age) | ADHD > Control | 0.50 | [-1.01, 2.01] | .507 | .938 | 0.011 | 0.20 |
| R_Precentral | DE_BDI_adj (Group+BDI+Age+Sex) | ADHD > Control | 0.72 | [-1.07, 2.51] | .422 | .784 | 0.017 | 0.30 |
| R_Precentral | DE_BDI_age (Group+BDI+Age) | ADHD > Control | 0.90 | [-0.93, 2.73] | .326 | .809 | 0.025 | 0.36 |
| R_Precuneus | TE_adj (Group+Age+Sex) | ADHD < Control | -2.11 | [-5.61, 1.39] | .230 | .967 | 0.037 | -0.49 |
| R_Precuneus | TE_age (Group+Age) | ADHD < Control | -1.77 | [-5.24, 1.70] | .308 | .938 | 0.026 | -0.41 |
| R_Precuneus | DE_BDI_adj (Group+BDI+Age+Sex) | ADHD < Control | -0.85 | [-4.12, 2.42] | .602 | .911 | 0.007 | -0.20 |
| R_Precuneus | DE_BDI_age (Group+BDI+Age) | ADHD < Control | -0.56 | [-3.81, 2.70] | .732 | .918 | 0.003 | -0.13 |
| R_Putamen | TE_adj (Group+Age+Sex) | ADHD > Control | 6.52 | [-1.85, 14.89] | .123 | .967 | 0.060 | 0.53 |
| R_Putamen | TE_age (Group+Age) | ADHD > Control | 6.31 | [-1.62, 14.25] | .116 | .938 | 0.061 | 0.51 |
| R_Putamen | DE_BDI_adj (Group+BDI+Age+Sex) | ADHD > Control | 8.14 | [-1.02, 17.31] | .080 | .675 | 0.078 | 0.65 |
| R_Putamen | DE_BDI_age (Group+BDI+Age) | ADHD > Control | 7.99 | [-0.79, 16.77] | .073 | .643 | 0.080 | 0.65 |
| R_RostralAnteriorCingulate | TE_adj (Group+Age+Sex) | ADHD > Control | 1.44 | [-4.56, 7.44] | .629 | .967 | 0.006 | 0.15 |
| R_RostralAnteriorCingulate | TE_age (Group+Age) | ADHD > Control | 1.86 | [-4.30, 8.02] | .544 | .938 | 0.009 | 0.19 |
| R_RostralAnteriorCingulate | DE_BDI_adj (Group+BDI+Age+Sex) | ADHD > Control | 2.75 | [-6.06, 11.57] | .531 | .863 | 0.010 | 0.28 |
| R_RostralAnteriorCingulate | DE_BDI_age (Group+BDI+Age) | ADHD > Control | 3.12 | [-5.94, 12.18] | .490 | .831 | 0.012 | 0.32 |
| R_RostralMiddleFrontal | TE_adj (Group+Age+Sex) | ADHD < Control | -0.53 | [-2.25, 1.20] | .541 | .967 | 0.010 | -0.20 |
| R_RostralMiddleFrontal | TE_age (Group+Age) | ADHD < Control | -0.36 | [-2.05, 1.33] | .672 | .938 | 0.005 | -0.13 |
| R_RostralMiddleFrontal | DE_BDI_adj (Group+BDI+Age+Sex) | ADHD < Control | -0.04 | [-1.90, 1.81] | .963 | .975 | 0.000 | -0.02 |
| R_RostralMiddleFrontal | DE_BDI_age (Group+BDI+Age) | ADHD > Control | 0.10 | [-1.73, 1.94] | .911 | .986 | 0.000 | 0.04 |
| R_SuperiorFrontal | TE_adj (Group+Age+Sex) | ADHD < Control | -0.66 | [-2.72, 1.41] | .523 | .967 | 0.012 | -0.23 |
| R_SuperiorFrontal | TE_age (Group+Age) | ADHD < Control | -0.57 | [-2.60, 1.46] | .573 | .938 | 0.009 | -0.20 |
| R_SuperiorFrontal | DE_BDI_adj (Group+BDI+Age+Sex) | ADHD > Control | 0.23 | [-1.96, 2.43] | .832 | .967 | 0.001 | 0.08 |
| R_SuperiorFrontal | DE_BDI_age (Group+BDI+Age) | ADHD > Control | 0.34 | [-1.86, 2.53] | .758 | .918 | 0.003 | 0.12 |
| R_SuperiorParietal | TE_adj (Group+Age+Sex) | ADHD < Control | -0.16 | [-1.91, 1.60] | .857 | .983 | 0.001 | -0.06 |
| R_SuperiorParietal | TE_age (Group+Age) | ADHD < Control | -0.15 | [-1.72, 1.42] | .846 | .962 | 0.001 | -0.06 |
| R_SuperiorParietal | DE_BDI_adj (Group+BDI+Age+Sex) | ADHD > Control | 0.52 | [-1.30, 2.33] | .568 | .897 | 0.009 | 0.20 |
| R_SuperiorParietal | DE_BDI_age (Group+BDI+Age) | ADHD > Control | 0.53 | [-1.13, 2.18] | .523 | .840 | 0.011 | 0.21 |
| R_SuperiorTemporal | TE_adj (Group+Age+Sex) | ADHD > Control | 0.68 | [-1.50, 2.87] | .532 | .967 | 0.010 | 0.22 |
| R_SuperiorTemporal | TE_age (Group+Age) | ADHD > Control | 0.71 | [-1.40, 2.82] | .500 | .938 | 0.011 | 0.23 |
| R_SuperiorTemporal | DE_BDI_adj (Group+BDI+Age+Sex) | ADHD > Control | 0.67 | [-1.76, 3.10] | .579 | .897 | 0.008 | 0.22 |
| R_SuperiorTemporal | DE_BDI_age (Group+BDI+Age) | ADHD > Control | 0.70 | [-1.67, 3.06] | .555 | .840 | 0.009 | 0.23 |
| R_Thalamus | TE_adj (Group+Age+Sex) | ADHD > Control | 3.67 | [-1.73, 9.07] | .177 | .967 | 0.048 | 0.55 |
| R_Thalamus | TE_age (Group+Age) | ADHD > Control | 3.69 | [-1.32, 8.69] | .145 | .938 | 0.054 | 0.56 |
| R_Thalamus | DE_BDI_adj (Group+BDI+Age+Sex) | ADHD > Control | 4.38 | [-1.22, 9.98] | .121 | .784 | 0.064 | 0.65 |
| R_Thalamus | DE_BDI_age (Group+BDI+Age) | ADHD > Control | 4.40 | [-0.89, 9.69] | .100 | .721 | 0.069 | 0.66 |
| R_TransverseTemporal | TE_adj (Group+Age+Sex) | ADHD > Control | 3.35 | [-7.61, 14.31] | .540 | .967 | 0.010 | 0.22 |
| R_TransverseTemporal | TE_age (Group+Age) | ADHD > Control | 2.79 | [-5.79, 11.37] | .515 | .938 | 0.011 | 0.18 |
| _TransverseTemporal | DE_BDI_adj (Group+BDI+Age+Sex) | ADHD > Control | 6.10 | [-9.61, 21.80] | .437 | .784 | 0.016 | 0.40 |
| R_TransverseTemporal | DE_BDI_age (Group+BDI+Age) | ADHD > Control | 5.67 | [-7.79, 19.12] | .399 | .809 | 0.018 | 0.37 |

Supplementary Table S2. Sensitivity power analysis (minimum detectable effects)

Notes. Minimum detectable effect sizes (MDES) were computed to quantify sensitivity for the models: TE_adj = Group + Age + Sex; TE_age = Group + Age; DE_BDI_adj = Group + BDI + Age + Sex; DE_BDI_age = Group + BDI + Age. For covariate-adjusted models, sensitivity is expressed as the minimum detectable incremental variance explained by the group term (partial R²), derived from Cohen’s f² for the F-test of the group coefficient (df1 = 1; df2 = N − p − 1, with p denoting the number of predictors in the model).

| **Model** | **Analysis / effect metric** | **α (two-sided)** | **Power** | **MDES (d or r)** | **MDES (partial R²)** |
| --- | --- | --- | --- | --- | --- |
| Two-group design | Two-group mean difference | 0.05 | 0.80 | 0.877 | — |
| Two-group design | FDR-corrected two-group mean difference | 0.00056 | 0.80 | 1.415 | — |
| TE_adj (Group + Age + Sex) | Incremental group effect in linear model (F-test; partial R² of group term) | 0.05 | 0.80 | — | 0.168 |
| TE_adj (Group + Age + Sex) | FDR-corrected incremental group effect in linear model (F-test; partial R²) | 0.00056 | 0.80 | — | 0.345 |
| TE_age (Group + Age) | Incremental group effect in linear model (F-test; partial R² of group term) | 0.05 | 0.80 | — | 0.164 |
| TE_age (Group + Age) | FDR-corrected incremental group effect in linear model (F-test; partial R²; Bonferroni α=0.05/89) | 0.00056 | 0.80 | — | 0.339 |
| DE_BDI_adj (Group + BDI + Age + Sex) | Incremental group effect in linear model (F-test; partial R² of group term) | 0.05 | 0.80 | — | 0.171 |
| DE_BDI_adj (Group + BDI + Age + Sex) | FDR-corrected incremental group effect in linear model (F-test; partial R²) | 0.00056 | 0.80 | — | 0.352 |
| DE_BDI_age (Group + BDI + Age) | Incremental group effect in linear model (F-test; partial R² of group term) | 0.05 | 0.80 | — | 0.168 |
| DE_BDI_age (Group + BDI + Age) | FDR-corrected incremental group effect in linear model (F-test; partial R²) | 0.00056 | 0.80 | — | 0.345 |
| Whole sample | Pearson correlation (two-sided; r) | 0.05 | 0.80 | 0.422 | — |
| Whole sample | FDR-corrected Pearson correlation (two-sided; r) | 0.00056 | 0.80 | 0.591 | — |
